# Neural precursors cells expanded in a 3D micro-engineered niche present enhanced therapeutic efficacy *in vivo*

**DOI:** 10.7150/ntno.50633

**Published:** 2021-01-01

**Authors:** Stephana Carelli, Toniella Giallongo, Federica Rey, Bianca Barzaghini, Tommaso Zandrini, Andrea Pulcinelli, Riccardo Nardomarino, Giulio Cerullo, Roberto Osellame, Cristina Cereda, Gian Vincenzo Zuccotti, Manuela Teresa Raimondi

**Affiliations:** 1 Pediatric Clinical Research Center “Romeo and Enrica Invernizzi”, L. Sacco Department of Biomedical and Clinical Sciences, University of Milano, Milano, 20157, Italy.; 2 Department of Chemistry, Materials and Chemical Engineering “Giulio Natta”, Politecnico di Milano, Milano, 20133, Italy.; 3 Istituto di Fotonica e Nanotecnologie (IFN)-CNR and Department of Physics, Politecnico di Milano, Milano, 20133, Italy.; 4 Genomic and Postgenomic Lab, IRCCS Mondino Foundation, Pavia, 27100, Italy.

**Keywords:** two-photon laser polymerization, pluripotency, nichoid, 3D micro-scaffolds, regenerative medicine

## Abstract

**Rationale:** Stem Cells (SCs) show a great potential in therapeutics for restoring and regenerating native tissues. The clinical translation of SCs therapies is currently hindered by the inability to expand SCs *in vitro* in large therapeutic dosages, while maintaining their safety and potency. The use of biomaterials allows for the generation of active biophysical signals for directing SCs fate through 3D micro-scaffolds, such as the one named “Nichoid”, fabricated with two-photon laser polymerization with a spatial resolution of 100 nm. The aims of this study were: i) to investigate the proliferation, differentiation and stemness properties of neural precursor cells (NPCs) following their cultivation inside the Nichoid micro-scaffold; ii) to assess the therapeutic effect and safety *in vivo* of NPCs cultivated in the Nichoid in a preclinical experimental model of Parkinson's Disease (PD).

**Methods:** Nichoids were fabricated by two photon laser polymerization onto circular glass coverslips using a home-made SZ2080 photoresist. NPCs were grown inside the Nichoid for 7 days, counted and characterized with RNA-Seq, Real Time PCR analysis, immunofluorescence and Western Blot. Then, NPCs were transplanted in a murine experimental model of PD, in which parkinsonism was induced by the intraperitoneal administration of the neurotoxin MPTP in C57/bl mice. The efficacy of engrafted Nichoid-expanded NPCs was evaluated by means of specific behavioral tests and, after animal sacrifice, with immunohistochemical studies in brain slices.

**Results:** NPCs grown inside the Nichoid show a significantly higher cell viability and proliferation than in standard culture conditions in suspension. Furthermore, we report the mechanical conditioning of NPCs in 3D micro-scaffolds, showing a significant increase in the expression of pluripotency genes. We also report that such mechanical reprogramming of NPCs produces an enhanced therapeutic effect in the *in vivo* model of PD. Recovery of PD symptoms was significantly increased when animals were treated with Nichoid-grown NPCs, and this is accompanied by the recovery of dopaminergic markers expression in the striatum of PD affected mice.

**Conclusion:** SCs demonstrated an increase in pluripotency potential when expanded inside the Nichoid, without the need of any genetic modification of cells, showing great promise for large-scale production of safe and functional cell therapies to be used in multiple clinical applications.

## Introduction

Stem cells (SCs) show a great potential in therapeutics for restoring and regenerating native tissues, due to their ability to commit to different types of functional cells^1^, ^2^. The development of therapies based on SCs is of key importance especially for diseases in which physiological tissue repair does not occur, including neurodegenerative diseases such as Parkinson's disease (PD)^3^, ^4^, spinal cord injuries^5^, ^6^ and amyotrophic lateral sclerosis^7^, ^8^. In these diseases, restorative therapies based on neural cell replacement using embryonic stem (ES) cell-derived neural cells have shown efficacy in animal models 9-12 but the clinical translation of therapies involving ES cells is generally opposed by medicines agencies, due to the risk of teratoma 13. To overcome these limitations, some of the authors developed a SCs therapy based on subventricular zone-derived neural progenitor cells (NPCs) 14, which proved effective in counteracting neurodegeneration in animal experimental models of traumatic spinal cord injury 5, 15 and PD 16-18. For a clinical translation of this therapy, it is necessary to increase SCs' survival in the unfavorable environment of neurodegeneration and, at the same time, their ability to promote both neuroregeneration and neuroprotection 9-12.

Nowadays, the approaches aiming at preserving stemness during cell expansion *ex vivo* are based on the use of substrates made up of feeder cells of animal origin or on the optimization of the culture media of SCs with a well-defined composition 19. Culture media also contain molecules, such as leukemia inhibitor factor (LIF) 20, that inhibit signaling pathways associated to cell death and differentiation, preventing the spontaneous change of cell phenotype during the *in vitro* expansions. Despite all the advantages offered by such chemical media conditioning, the long-term side-effects of soluble factors for SCs culture on humans would be risky and difficult to assess 21. These criticalities highlight the translational inapplicability of the available expansion methods, although they are effective in maintaining stemness. The interest has recently been shifted towards mechanical stimuli as an instrument for *in vitro* regulation of SCs characteristics. Moreover, mechanical stimuli may be sufficient *in vitro* for the control of the proliferative and differentiation potential of SCs 22, 23.

Several studies have shown that passive mechanical stimuli, given by the micro and nano-topography of the substrate, stiffness and three-dimensional (3D) microarchitecture, are able to modulate the behavior of SCs grown *in vitro*, even in the absence of biochemical stimuli 24, 25. Control of the scaffold structural properties, i.e. the 3D architecture combined to the material stiffness, plays a key role in the determination of SCs' fate 22, as it enables to determine the configuration and the level of the forces exchanged between the extracellular environment and the focal adhesion sites, which activate the cell transduction pathways regulating SCs' activities^22^, ^26^. A crucial limitation of most of the currently available approaches for 3D substrate fabrication, as solvent casting, particulate leaching, gas foaming, electrospinning and fiber bonding, is that they do not allow the control of the substrate's 3D geometrical structure on a length scale comparable to the cell size 27, 28. To overcome these limitations, some of the authors used the high precision direct laser writing technique called two-photon laser polymerization (2PP) 29, 30 to develop a 3D micro-engineered culture substrate named “Nichoid” 31, 32 able to maintain the stemness capacity of primary rat mesenchymal stem cells (MSCs), human bone marrow-derived MSCs and mouse ES cells 22, 33-35.

Here we report that these engineered niches are able to reprogram the stemness features of adult NPCs without the need of exogenous chemical factors, by exclusively controlling the 3D structural properties of the cell adhesion microenvironment during cell expansion. In addition, we show that the therapeutic dose of NPCs can be reduced by two thirds with the same efficacy, compared to cells cultured in conventional spheroids, in an extensively characterized pre-clinical experimental mouse model of PD. Since the main obstacle to the entry of SCs into clinical practice is the lack of specific *in vitro* culture methods able to guarantee large-scale expansion while maintaining the cells safety, potency and function 36, our findings may have a tremendous impact in the clinics, opening a new perspective of production of large amounts of safe and effective NPCs for use in regenerative therapies of the nervous system.

## Methods

### Fabrication of the Nichoids by two-photon laser polymerization (2PP)

The details of the 2PP fabrication method have been previously reported 29. Briefly, ultrashort laser pulses from an infrared femtosecond laser (femtoRegen, HighQ laser, 8 W maximum average power, 400 fs pulses, 1042 nm wavelength) are focused by a high numerical aperture microscope objective (100×, 1.4 numerical aperture, oil immersion, Zeiss) inside the volume of a UV-photosensitive resin. The material chosen for the present work is a hybrid organic-inorganic photoresist based on Silicon and Zirconium, specifically designed for 2PP 37, 38, which is mixed with a commercial photoinitiator, Irgacure-369 (2-Benzyl-2-dimethylamino-1-(4-morpholinophenyl)-butanone-1). The sample is translated with respect to the laser beam by fast and high-precision linear stages (ANT-130, Aerotech) that enable the direct writing of the three-dimensional microstructure. After the irradiation process, a development step is performed in a 50:50 solution in volume of 4-methyl-2pentanone and isopropyl alcohol, where the unirradiated resin is washed away leaving the irradiated polymeric 3D structure. In order to speed-up the fabrication process, a parallel multi-foci 2PP process has been implemented, with the use of a liquid-crystal phase-only spatial light modulator (PLUTO NIR-049, Holoeye). This allowed drawing simultaneously up to six lines, with balanced power between the different foci, at 3 mm/s translation velocity. The fabricated structures were inspected with a table-top scanning electron microscope (Phenom PRO, Phenom-World), in order to control the fabrication quality and to verify the line size (Figure S1).

### Preparation of the Nichoid substrate

The 2PP-patterned substrates (Nichoid) were washed thoroughly, kept for 20 minutes in deionized water, disinfected for 90 minutes in 70% ethanol (VWR), washed repeatedly in sterile deionized water and dried under UV for 90 minutes in sterile conditions.

### Primary cells isolation and differentiation

NPCs expressing green fluorescent protein (GFP) were isolated 6 h postmortem from adult C57BL/6-Tg(UBC-GFP)30Scha/J mice weighing 25-30 g (Charles River) as previously described^3^, ^14^-^16^. NPCs were maintained in culture in Neurobasal Medium (GIBCO^TM^, Life Technologies) containing 2% B-27 supplement (Life Technologies), 1% L-Glutamine (Euroclone), 1% penicillin and streptomycin (Euroclone), b-FGF (human recombinant, 20 ng/mL) and hEGF (human recombinant, 20 ng/mL)^14^, ^15^, ^39^, ^40^. After one week, these cells gave rise to floating neurospheres in culture (i.e. suspension culture conditions) with a mean diameter of 100 - 200 μm^41^, ^42^. The spheroids formed were harvested, collected by centrifugation (10 minutes at 123xg), mechanically dissociated by pipetting to a single cell suspension and re-plated in medium at the density of 1×10^4^ cells/cm^2^.

In order to verify the multipotency of NPCs, cells were subjected to *in vitro* differentiation as already reported 14, 15, 39. Briefly, neurospheres were mechanically dissociated and seeded at the density of 1.5×10^4^ cells/well in the two different experimental conditions, either on a flat glass in presence of an adhesive substrate (Matrigel™, BD Biosciences; control condition) or inside the Nichoid scaffold without an additional adhesive substrate. Cells were grown for 48h in presence of NPCs medium with bFGF (10 ng/mL). The differentiation medium was then substituted with NPCs medium containing 2% FBS (Life Technologies) without growth factors for 5 days 14-16. The expression of different neural markers was investigated by immunofluorescence in the SVZ of adult mouse brain, from which NPCs were derived. Several of these markers result notably expressed in this brain area (Figure S2A): Nestin, a stemness marker of neural cells, Glial Fibrillary Acidic Protein (GFAP), an astrocyte marker, Microtubule Associated Protein 2 (MAP2) and Doublecortin (DCX), markers of NPCs (Figure S2A). With the aim to monitor the presence of NPCs post-transplantation, NPCs were derived from the mice strain C57BL/6-Tg (UBC-GFP) 30Scha/J mice which express GFP under the ubiquitin promoter 3, 18, 43. Consequently, NPCs express the Green Fluorescent Protein (GFP NPCs). In agreement with our previous observations 14, GFP NPCs are also able to form spheroids and have the same growth capability as NPCs (Figure S2B). Neurospheres obtained from GFP NPCs also present a strong expression of Nestin in the center of the structure and MAP2 more distributed in the external area, as expected 14, 44 (Figure S2C). To determine that GFP expression does not alter NPCs features, GFP NPCs were then differentiated for 8 days and the expression of Nestin, MAP2, GFAP and NG2 (chondroitin- sulfate proteoglycan) was evaluated by immunofluorescence (Figure S2D) 14.

### Cell seeding in the Nichoid

Cells were cultured inside the Nichoid to evaluate its effect on proliferation and differentiation. The cells grown in culture in floating conditions were harvested, collected by centrifugation (123×g for 10 minutes), mechanically dissociated and counted with trypan blue exclusion method (Sigma Aldrich). 1×10^4^ cells were seeded at the center of the Nichoid in a single drop of NPCs medium. The multi-well was kept in the incubator at 37°C and 5%CO_2_ for 1 hour to allow cells to enter the 3D niches and then 465 μL of NPCs medium were added.

### Cells' detachment from the Nichoid

In order to collect NPCs, cells were washed with PBS (Life Technologies) and detached with 200 μL of Citric Saline Solution administrated for 10 minutes. Citric Saline Solution was prepared using 0,135 M KCl (Fluka BioChemika) and 0.015 M Sodium citrate dihydrate (Sigma Aldrich). Cells were then collected in NPCs medium and pelleted (123×g for 10 min).

### Cellular proliferation assay

Cells were counted using the trypan blue exclusion method. Specifically, cells were incubated with trypan blue, transferred in a Bürker chamber and examined by light microscopy and scored as able (live) or unable to exclude the dye (dead).

### Scanning Electron Microscope

For the environmental scanning electron microscopy (ESEM) the samples were fixed by dehydration with ethanol. ESEM operates in a low vacuum mode, and this condition improves the images resolution. In order to prepare the sample for the analysis, the culture medium was removed and cells were incubated overnight at 4°C with a solution composed of 1.5% (v/v) glutaraldehyde 50% (v/v) in water and 0.1 M sodium cacodylate (pH=7.1-7.2). Then, samples were rinsed with 0.1 M sodium cacodylate buffer and incubated for 5 min each with increasing ethanol concentrations (20%, 30%, 40%, 50%, 60%, 70%, 80%, 90%, 96% and 100% v/v). This passage was repeated twice. The images were acquired using the ESEM ZEISS EVO 50 EP.

### RNA extraction

Total RNA from cultured cells was isolated using TRIzol Reagent (Invitrogen) following standard protocol. RNA quality was assessed using a spectrophotometer (NANOPhotometer® NP80, IMPLEN) and a 2100 Bioanalyzer (Agilent RNA 6000 Nano Kit, Waldbronn, Germany); RNAs with a 260:280 ratio of ≥1.5 and an RNA integrity number of ≥8 were subjected to deep sequencing.

### Libraries preparation for RNA-Seq and bioinformatic data analysis

Sequencing libraries were prepared with TruSeq Stranded Total RNA kit (Illumina) using 200ng total RNA. Qualities of sequencing libraries were assessed with 4200 TapeStation with the DNA1000 reagent kit. RNA processing was carried out using Illumina NextSeq 500 Sequencing. FastQ files were generated via Illumina bcl2fastq2 (Version 2.17.1.14 - https://support.illumina.com/downloads/bcl2fastq-conversion-software-v2-20.html) starting from raw sequencing reads produced by Illumina NextSeq sequencer. Qualities of individual sequences were evaluated using FastQC software (see Code Availability 1) after adapter trimming with cutadapt software. Gene and transcript intensities were computed using STAR/RSEM software 45 using Gencode Release 27 (GRCh38) as a reference, using the “-strandness forward” option. Differential expression analysis for mRNA was performed using R package DESeq2 46, selected because of its superior performance in identifying isoforms differential expression 47. Genes were considered differentially expressed and retained for further analysis with |log_2_(nichoid sample/control sample)| ≥ 1 and a FDR ≤ 0.1. We imposed minimum |Log_2_FC| of 1 and a FDR lower than 0.1 as thresholds to differentially expressed genes. This choice is motivated by the decision to maximize the sensitivity of this analysis, in order to perform a massive screening and identify candidate genes to be validated with a wider sample population with real-time analysis. The raw data obtained from the RNA-seq analysis is deposed on the Gene Expression Omnibus repository (GSE150767).

### Pathway analysis

We performed a Kegg pathway analysis (Kyoto Encyclopedia of Genes and Genomes http://www.genome.ad.jp/kegg) and a WikiPathways analysis of differentially expressed coding genes via enrichR web tool 48, 49. The R software was used to generate Dotplot graphs (with the ggplot2 library) and Pathview graphs (with the Pathview library 50).

### Real Time PCR

Total RNA (500 ng) was reverse transcribed using iScript cDNA synthesis kit (Bio-Rad) according to the manufacturer's instructions. Using gene sequences available from NCBI for target genes http://www.ncbi.nlm.nih.gov/nucleotide, PCR oligonucleotide primers for target genes were selected and primers sequences are reported in Table S1 This was done using the NCBI's Primer-BLAST tool. Real-Time PCR was performed with StepOnePlus^TM^ Real-Time PCR System (Thermo Fisher) using SSO SYBR Green Supermix (Bio-Rad). Genes were quantified in triplicates and GAPDH was used as housekeeping gene. Gene expression was calculated using the 2^-ΔΔCt^ method.

### Methylation

Total DNA was extracted using FitAmp Blood and Culture Cell DNA Extraction Kit (Epigentek) in accordance with manufacturer's instructions. Successively, the MethylFlash^TM^ Methylated DNA Quantification Kit Colorimetric (Epigentek) was used for detecting global DNA methylation status. The amount of DNA for each assay used was 100 ng. The absorbance was determined with the Ensight™ multimode plate reader (PerkinElmer) at 450 nm. To determine the specific methylation status of each DNA sample (Floating vs Nichoid expansion approach), the calculation of percentage of 5-mC in total DNA was carried out using the following formula:


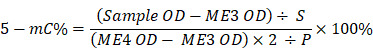


where S is the amount of input sample DNA in ng (100), P is the amount of input positive control in ng, ME4OD is the absorbance of the positive control and ME3 OD is the absorbance of the negative control.

### Western Blot

The NPCs detached from Nichoid and cells grown in standard floating condition were collected by centrifugation and washed with PBS 0.1 M pH 7.4 (Life Technologies). Cell protein extracts were obtained with a lysis buffer composed of Tris-HCl pH 6.8, SDS 1%, Na AC 0.1M and EDTA 5 mM with 1 μl/ml of a proteases inhibitor cocktail (Sigma Aldrich). 800 μl of Lysis buffer was added to cell pellets, which were then frozen in dry ice and defrosted on the thermic plate for three times 51.

Aliquots of each sample containing equal amount of proteins (30 μg), were separated by SDS-PAGE and transferred onto Nitrocellulose membranes. Membranes were then blocked in 5% slim milk (diluted in TBS with 0.05% Tween-20) and probed with the appropriate primary antibody against NESTIN (monocl. 1:200; Millipore MAB353-2444141), BETA-TUBILIN III (BETA-TUB III; monocl. 1:1000 GeneTex GTX631836), NANOG (monocl. 1:1000 GeneTex GTX100863), OCT4 (monocl, 1:1000 Cells Signaling 2840), SOX2 (monocl. 1:5000 GeneTex GTX101507), and beta-actin (monocl 1:1000; Sigma-Aldrich A1978) overnight at 4°C.

The membrane was then incubated with specific secondary antibody Anti-Rabbit/Mouse IgG HRP (1:5000 dilution; Jackson Immuno Research Laboratories) Proteins were visualized by means of an enhanced chemiluminescence detection system (ECL™, Amersham, Arlington Heights, IL). After acquisition by a GelDoc^TM^ image capture system (Kodak), the proteins present on the nitrocellulose membrane were quantified using the ImageJ software using beta-actin as an internal control.

### Immunocytochemistry analysis

At the end of specific treatment cells were fixed with 4% paraformaldehyde (BDH) in PBS 0.1 M pH 7.4 (Life Technologies) for 10 min at room temperature, then for another 10 minutes in 2% paraformaldehyde and washed with PBS. Samples were incubated overnight at 4 °C in PBS containing 10% normal horse serum (NHS, Thermo Fisher), 0.3% Triton X-100 (BDH), and the appropriate primary antibody. Cells' characteristics were assessed by immunocytochemistry with antibodies against Microtubule Associated Protein 2 (MAP2, polycl. 1:200; Millipore AB5622) Glial Fibrillary Acidic Protein (GFAP, polycl. 1:1000; Covance PRB571c), Nestin (monocl. 1:200; Millipore MAB353-2444141), BETA-TUBILIN III (BETA-TUB III; monocl. 1:500/20 000 GeneTex GTX631836), Chondroitin sulfate proteoglycan (NG2, 1:200; Millipore AB5320). After thorough washing with PBS and 10% NGS, cells were incubated for 90 min at room temperature with the appropriate secondary antibody (Alexa Fluor® 488 and 546, Life Technologies). Nuclei were stained with 4′,6-diamidino-2-phenylindole (DAPI 1µg/ml) (Sigma Aldrich), 10 minutes at room temperature, mounted using the FluorSave Reagent (Calbiochem, Merck Chemical), and analyzed by confocal microscopy (Confocal laser scanning microscopy Olympus Fluoview FV10i). The ImageJ software was used for microphotographic digital analysis. In control determinations, primary antibody was omitted and replaced with equivalent concentrations of unrelated IgG of the same subclass. The positive pixels were quantified against the negative background. The microscope light intensity of the laser was the same for all analyzed sections and for determining the background optical density.

### Animals and study approval

Procedures involving animals and their care were conducted in conformity with the Italian Guidelines for Laboratory Animals, which conform to the European Communities Directive of September 2010 (2010/63/UE) and the Review Committee of the University of Milan gave its approval to the study (N° 778/2017). Male C57BL/6 mice (Charles River, Milan, Italy), 12-16 weeks old and weighing 20-24 g were kept for at least 7 days before the experiments in standard conditions (22 ± 2 °C, 65% humidity, and a 12h light-dark cycle) with food and water ad libitum. Moreover, in order to make them amenable the animals were accustomed to the behavioral tests (horizontal and vertical, see below for details) daily for one week prior to 1-methyl-4-phenyl-1,2,3,6-tetrahydropyridine (MPTP) i.p. injection.

### Animal treatments

Experimental animals were divided into four groups: 1) Control (healthy animals, n = 6); 2) MPTP treated mice (MPTP, n = 6); 3) MPTP treated mice transplanted with NPCs grown in standard conditions (MPTP + GFP NPCs n = 6); 4) MPTP treated mice transplanted with NPCs grown inside the Nichoid (MPTP + GFP NPCs grown on Nichoid, n = 6). Parkinsonism was induced by intraperitoneal (i.p.) administration of MPTP in C57BL/6 mice following the acute paradigm with a small modification. Briefly, animals were administered a double dose of MPTP hydrochloride: a first i.p. injection of MPTP (36 mg/kg) and a second i.p. injection of MPTP (20 mg/kg) after 7 days. GFP NPCs treated animals were transplanted with 5x10^4^ cells/μl (5 μl) GFP expressing NPCs grown in standard condition, and with 1.4x10^4^ cells/μl (5 μl) grown inside the Nichoid, according to the following stereotaxic coordinates in relation to bregma: 0.1 mm posterior, 2.4 mm mediolateral and 3.6 mm dorsolateral the level of left striatum 3, 18, 43.

### Behavioral tests

To investigate the recovery of motor dysfunction after cell transplantation two different behavioral tests were performed: horizontal and vertical grid tests 18, 43, 52.

### Horizontal grid test

The grid apparatus was constructed according to Tillerson and co-workers 18, 43, 52. The animal was videotaped for 30s and the videos were replayed for percentage forepaw fault analysis using a recorder with slow motion option. The number of unsuccessful forepaw steps divided by the total number of attempted forepaw steps was evaluated 52. Three observers, in blind, rated each trial for forepaw faults per step.

### Vertical grid test

The vertical grid apparatus was constructed according to Kim and co-workers 18, 43. For this test, the mouse was placed 3 cm from the top of the apparatus, facing upwards, and was videotaped when turning around and climbing down. The score reported was the time required by the mouse to make a turn, climb down and reach the bottom of the grid with its forepaw within 180 s 18, 43. The analysis was performed by three observers in blind.

### Immunohistochemistry

For immunohistochemistry analyses, animals from each group were anesthetized by i.p. injection of sodium pentobarbital (65 mg/kg), perfused by trans-cardiac perfusion with 50 mL of saline solution and fixed with 200 mL of 4% paraformaldehyde in 0.1 mol/L PBS.

Brains were dissected and post-fixed overnight in the same fixative, cryoprotected with 30% sucrose (Sigma Aldrich), quickly frozen, stored at -80°C, and sectioned by means of a cryostat (CM 1850, Leica Microsystems). Sections (20µm) were rinsed with PBS, treated with blocking solution (Life Technologies) and incubated with the appropriate primary antibody with antibodies against Tyrosine Hydroxilase (TH, monocl. 1:500; Sigma Aldrich T2928); Dopamine Transporter (DAT, monocl. 1:1000; Millipore MAB369) Glial Fibrillary Acidic Protein (GFAP, polycl. 1:1000; Covance PRB571c); Monocite/Macrophages (MOMA, monocl. 1:25; Millipore MAB1852) overnight at 4°C. The sections were then washed with PBS and incubated with appropriate secondary antibodies (Alexa Fluor® 488 and 546, Life Technologies) for 2 hours at room temperature. Sections were then washed in PBS, nuclei were stained with DAPI (1µg/ml final concentration, 10 minutes at room temperature), mounted using the FluorSave Reagent (Calbiochem, Merck Chemical), and analyzed by confocal microscopy (Confocal laser scanning microscopy Olympus Fluoview FV10i). The ImageJ software was used for microphotographic digital analysis. The positive pixels were quantified against the negative background eliciting an index score which includes fibers and neuronal bodies. The microscope light intensity of the laser was the same for all analyzed sections and for determining the background optical density. For the quantification of positive cells, the number of cells positive to the staining was counted with respect to the total nuclei number.

### Statistical analysis

Data is expressed as mean ± SD. When only two value sets were compared, the statistical analysis was performed with Student's t-test. When three or more value sets were analyzed one-way ANOVA was used and Bonferroni's post-test were applied. The Prism 7 software (GraphPad Software Inc., La Jolla, CA) was used assuming a *P* value less than 0.05 as the limit of significance. Behavioral data were analyzed with a two-way ANOVA test followed by Bonferroni post-test.

## Results

### Nichoids fabrication by multi-foci 2PP

2PP is like a 3D printer at the micro/nanoscale. Exploiting nonlinear absorption of ultrashort pulses in photosensitive resins, it can induce photopolymerization in very small volumes, only at the laser beam focus. Translation of the sample with respect to the beam allows writing arbitrary 3D structures in the volume of the resin, like the Nichoid 32. The hybrid organic-inorganic photoresist used to structure the Nichoid, SZ2080 32, 37, with a Young's modulus around 0.14 GPa and chemically inert, has been already extensively validated for SCs culture 31, 34, 35. The fabricated Nichoids were analyzed by 3D Scanning Electron Microscopy to investigate the quality of the sample (Figure S1A). The single niche is a block of 90x90 µm and 30 µm height. This geometry was repeated to obtain a block of 450×450 µm (Figure S1A). For the biological validation, we used a supermatrix obtained by repetitions of the above reported blocks covering an area of flat glass of 8 mm in diameter. This allowed limiting the extension of the flat glass area surrounding the engineered niches to a circular ring of 2 mm in width. An optimized and faster microfabrication protocol was applied to obtain the engineered micro-niches, using a spatial light modulator to shape the laser beam in order to produce multiple and properly spaced foci after the objective 29 (Figure S1B). In this way, up to six parallel lines can be written in a single scan, thus significantly reducing the production time of the complex mesh of Nichoids over large surfaces. Typical features of the Nichoid are a porosity of 91% and a regular and extended structure, which allows its application as a scaffold for cell culture (Figure S1A enlargement). All the Nichoids used for the biological experiments, reported in the following sections, have the above described geometrical features.

### NPCs proliferate inside the Nichoid and express stemness markers

**Figure 1:**
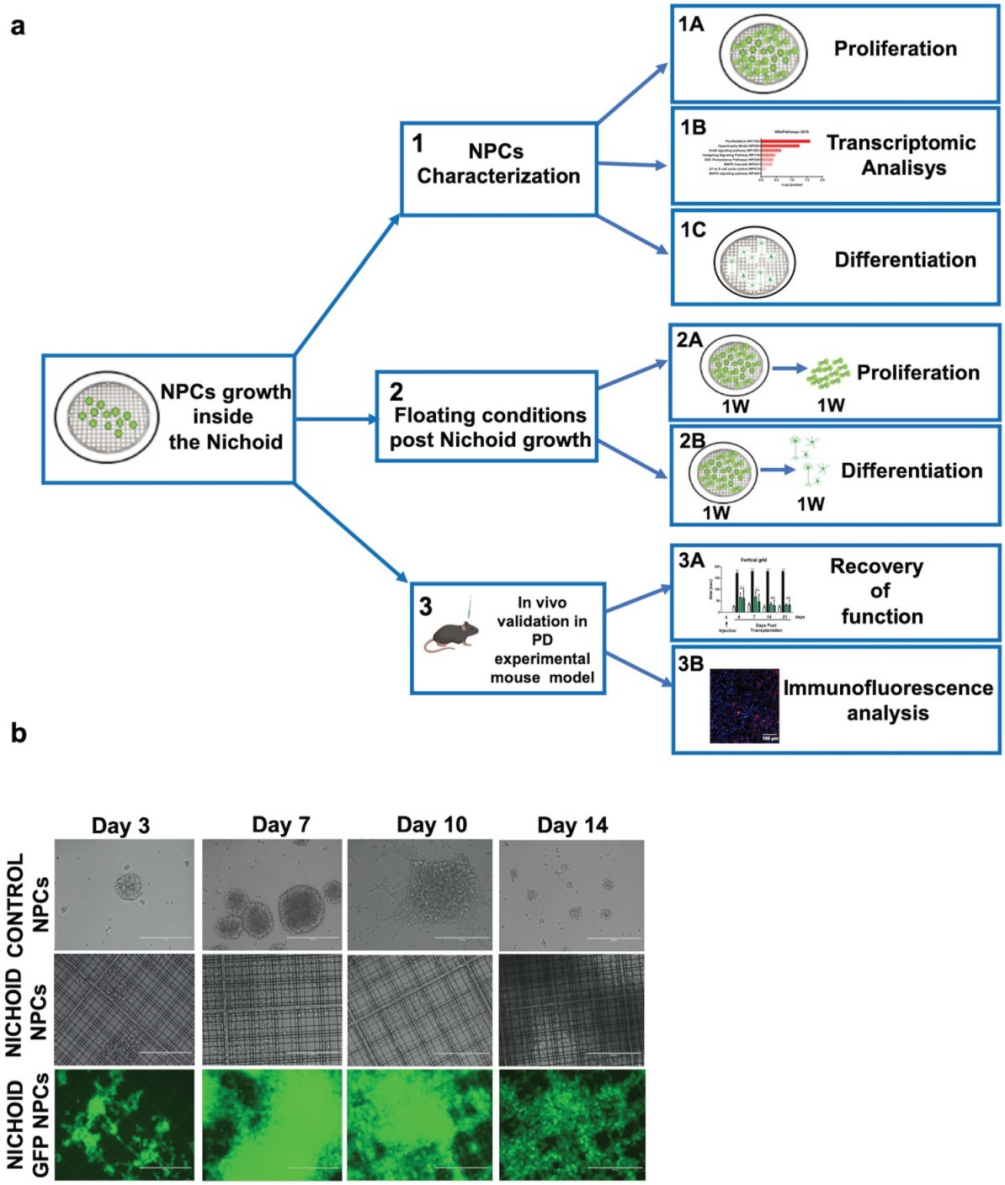

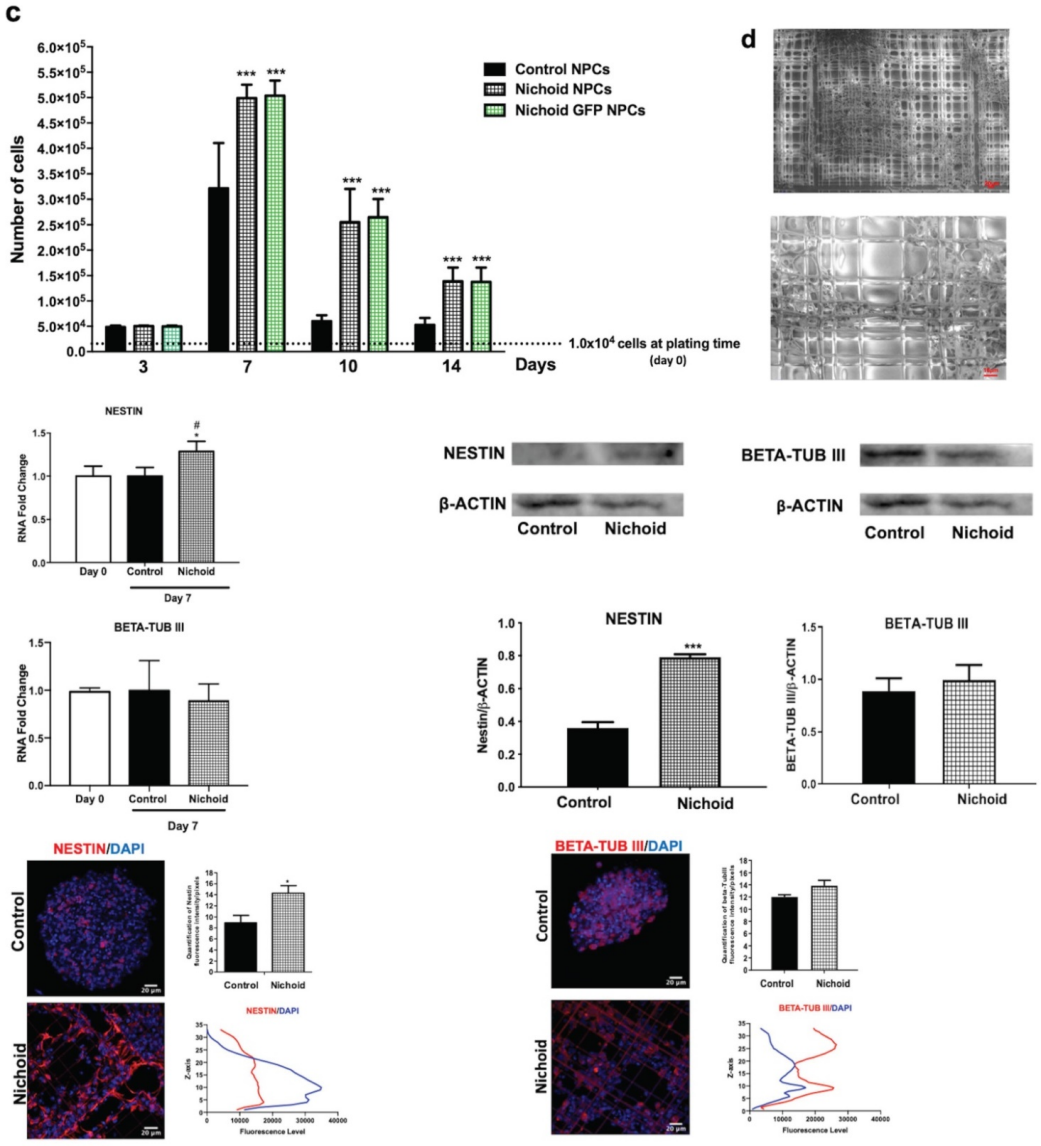
** Proliferation and neural features of NPCs expanded inside the Nichoid. A.** Experimental set up of the study. **B.** Representative *in vivo* light images of NPCs and GFP-NPCs grown in standard conditions (Control NPCs) or inside the Nichoid (Nichoid NPCs; Nichoid GFP NPCs) at day 3, 7, 10, 14. Scale bars 200 µm. **C.** Proliferation analysis of NPCs and GFP NPCs grown inside the Nichoid or in standard conditions. 1x10^4^ cells were plated at day 0 and counted with trypan blue exclusion method. Data are expressed as mean ±SD (n=6, ***p<0.001 vs Control NPCs). **D.** Representative images of Nichoid-grown NPCs analyzed by Environmental Scanning Electron Microscope (ESEM). Scale bars 20 µm and 10 µm. **E.** mRNA expression levels of Nestin and beta-TubIII of NPCs at day 0 and maintained inside the Nichoid or in standard conditions for 7 days. Results are expressed as mean ±SD (n=3; *p < 0.05 vs Control; #*p*<0.05 vs Day 0). **F.** Representative immunoblot images of NESTIN and BETA-TUBIII in NPCs maintained for 7 days inside the Nichoid or in standard conditions (n=2 for NESTIN and n=3 for BETA-TUBIII). Band intensity quantification was measured with ImageJ software. Data correspond to the mean ± SD (***p <0.001 vs Control). **G.** Representative immunofluorescence images of NESTIN and BETA-TUBIII in NPCs grown inside the Nichoid for 7 days or in standard conditions. Nuclei were stained with DAPI. Scale bars: 20 µm. Fluorescence intensity was quantified with ImageJ software. Data corresponds to mean ± SD (3 fields/3 independent experiments, n=9, *p < 0.05 vs Control). The lower panels show the markers' distribution along the Z axis.

The experimental setup used in this study is depicted in panel A of Figure 1. As shown in Figure 1B and Figure S3A, NPCs in standard conditions start forming neurospheres in suspension culture, reaching a mean diameter of 100-200 µm after 7 days in culture. At following time points, the spheres reach an excessive dimension, which does not allow the cells to receive enough nutrients, leading to an increase in cell death and to a decrease in spheres' dimension (Figure 1B and 1C and Figure S3B).

To evaluate the proliferation capabilities of NPCs and NPCs physiologically expressing the green fluorescent protein (GFP NPCs) inside the Nichoid compared to standard floating conditions, 1x10^4^ cells were plated at day 0 of the experiment and let grow, in standard NPCs medium, for different time points for up to 14 days (Figure 1B, 1C and Figure S3). When NPCs and GFP NPCs were grown inside the Nichoid, they did not form neurospheres as in normal floating conditions; instead, the cells created a flat aggregate resembling a carpet, expanding inside the niches (Figure 1B and 1D and Figure S3A). Cells were counted at day 3, 7, 10 and 14 (see M&M section for further details), reaching their maximum proliferation at day 7 (Figure 1C and Figure S3B). These results led us to use the 7 days' time point for further experiments (Figure 1A). The panel D of Figure 1 reports the analysis performed by Environmental Scanning Electron Microscopy of NPCs grown inside the Nichoid for 7 days. These high-resolution images allowed for an appreciation of how the cells interact with the surrounding scaffold, forming tight connections with the Nichoid grid.

After 7 days NPCs expansion we investigated the mRNA's expression of Nestin and beta-tub III, two well defined neural markers 53. Figure 1E shows that NPCs' maintenance inside the Nichoid determines the increase in the mRNA of the stemness marker Nestin. On the other hand, the expression of beta-tub III, a marker of fully differentiated neurons, is as expected not significantly different from the standard floating conditions (Figure 1E).

Western Blot and Immunofluorescence analysis (Figure 1F,G and Figure S4) confirmed that the expression of Nestin is significantly enhanced in NPCs grown inside the Nichoid compared to those in standard floating conditions, while beta-tub III's expression was not affected. Moreover, the analysis of the specific markers distribution inside the Nichoid, with respect to the vertical (Z) axis (Figure 1G), revealed that Nestin positive cells were mainly localized at the bottom and center of the synthetic niches (Figure 1G left). Beta-tub III, on the other hand, appears to be mainly expressed by the cells found at the external layers of the niches (Figure 1G right).

### RNA-sequencing analysis reveals multiple pathways involved in pluripotency

**Figure 2:**
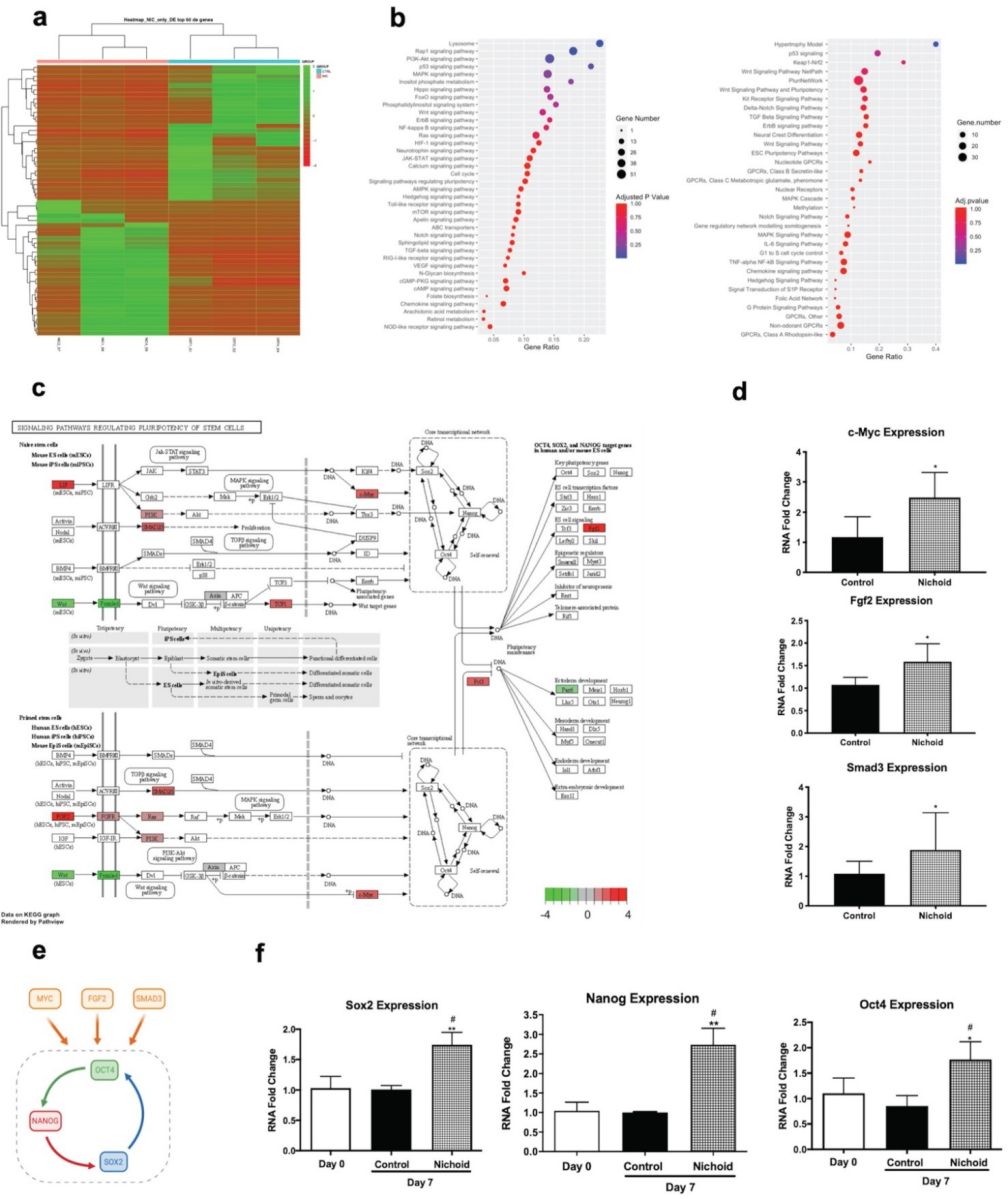
** The Nichoid modifies gene expression inducing pluripotency. A.** Expression profiles of differently expressed genes in Nichoid-grown NPCs vs standard conditions reported as a Heatmap. We considered as differentially expressed only genes showing |log2(Nichoid samples/control samples)| ≥ 1 and a False Discovery Rate ≤ 0.1. **B.** Dot plot graph of KEGG 2019 and WikiPathways 2019 analysis reports significantly deregulated pathways correlated to pluripotency in Nichoid-grown NPCs. The dot's dimension corresponds to the number of genes implicated in each pathway and the color refers to pathway's significance. **C.** Pathview analysis of signaling pathways regulating pluripotency of stem cells in Nichoid-grown NPCs vs standard conditions. Genes implicated are colored in the pathways, and the color-scale refers to the gene's fold change deregulation. **D.** c-Myc, Fgf2 and Smad3 mRNA expression levels in Nichoid-grown NPCs versus control. Results are expressed as mean ± SD of three independent experiments performed in duplicate (n=6; *p < 0.05; **p<0.01 vs Control). **E.** Schematic representation of three genes identified with transcriptome analysis (c-Myc, Fgf2 and Smad3) and their activation of pluripotency genes Oct4, Nanog and Sox2. Made in ©BioRender-biorender.com. **F.** Sox2, Oct4 and Nanog mRNA expression levels at day 0 and in Nichoid-grown NPCs versus control. Results are expressed as mean ± SD of three independent experiments performed in duplicate (n=6; *p < 0.05; **p<0.01 vs Control; #*p* < 0.05 vs NPCs at day 0).

**Figure 3:**
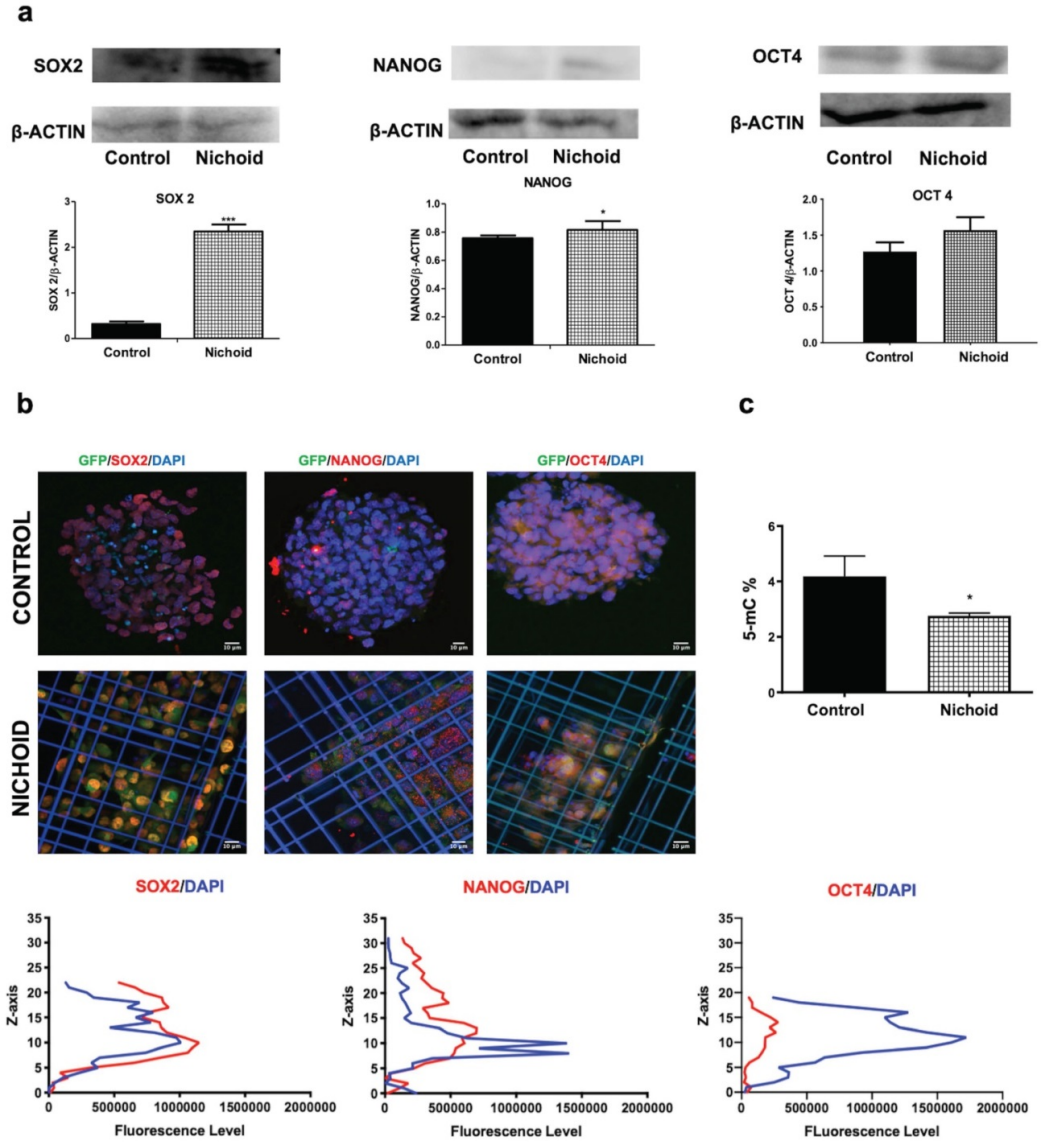
** Induction of protein expression of pluripotency core-network and hypo-methylation in Nichoid-grown NPCs. A.** Representative immunoblot images of western blot analysis of SOX2, NANOG and OCT4 in Nichoid-grown NPCs versus control. ß-Actin was used as loading control (n=3). The histograms report the band intensity quantifications, measured with ImageJ software. Data correspond to mean ± SD (*p<0.05; ***p <0.001 vs Control). **B.** Immunofluorescence analysis of SOX2 (red), NANOG (red) and OCT4 (red) of Nichoid grown NPCs versus control. Nuclei are stained in blue (DAPI). The lower panels show the markers' fluorescence distribution along the Z axis. **C.** Total DNA methylation of Nichoid-grown NPCs versus control. Results are expressed as mean ± SD (*p <0.05 vs Control).

In order to investigate the effects of expansion inside the 3D niche on NPCs, we performed a deep sequencing transcriptomic analysis. Specifically, NPCs grown inside the Nichoid for 7 days compared to standard conditions in suspension were subjected to RNA-sequencing. Heatmap analysis shows different expression profiles in the two conditions, with NPCs grown in standard floating condition and Nichoid-grown NPCs spontaneously grouping (Figure 2A). The analysis identified 1934 deregulated transcripts, suggesting a profound impact of the Nichoid on cellular transcription (Figure 2A). Amongst the significantly deregulated pathways, evaluated with Kyoto Encyclopedia of Genes and Genomes (KEGG) analysis and WikiPathways, it was interesting to observe that a large number of them correlated with both pluripotency and cellular proliferation (Figure 2B). Specifically, out of the 277 pathways obtained after KEGG analysis 38 resulted correlated to pluripotency. This was confirmed also by WikiPathways where 33 out of the 149 obtained pathways were correlated to pluripotency (Figure 2B). This suggests that the Nichoid could increase the pluripotency capabilities of NPCs by up-regulating key genetic regulators of this process (Table S2). To fully eviscerate this concept, pathview analysis of “signaling pathways regulating pluripotency of stem cells” allowed the identification of key genes involved in this process, along with their fold change deregulation (Figure 2C). Amongst these, we noted the presence of c-Myc, Smad3 and Fgf2, key actors in the pluripotency pathways found up-regulated in NPCs grown inside the Nichoid, and we assessed their increased expression also by Real Time PCR (Figure 2C and 2D). These three transcription factors and their downstream target genes coordinately promote self-renewal and pluripotency 54-56. Indeed, all these genes converge to the activation of a core transcriptional network involving SOX2, NANOG and OCT4, also found up-regulated in NPCs expanded inside the Nichoid (Figure 2E, Figure 2F). These factors are expressed at high levels in embryonic stem cells 57. Moreover, they regulate the expression of other genes during development and are found at high levels in pluripotent stem cells 58-60. The expression of SOX2, NANOG and OCT4 was also investigated in the above reported experimental conditions through Western blot and immunofluorescence analysis, confirming that their expression at protein level is significantly enhanced (Figure 3A, Figure 3B and Figure S4). The distribution of the three markers described above (Figure 3B) showed that NPCs positive to SOX2, NANOG and OCT4 staining are mainly distributed in the lower and central part of the Nichoid, suggesting a similarity with a biological niche.

Gene expression potential in SCs renewal and differentiation could be regulated by epigenetic processes, of which DNA methylation is the most characterized 61. To examine the chromatin status of NPCs expanded inside the 3D niche, compared to standard floating conditions, we evaluated the global DNA methylation levels, which decreased in cells grown inside the Nichoid with respect to controls (Figure 3C). This is a specific characteristic of multipotent SCs 62 and represents an additional evidence of the relevance of the Nichoid in the enhancement of pluri/multipotency capabilities without exogenous factors.

### NPCs keep memory of their growth inside the Nichoid

**Figure 4:**
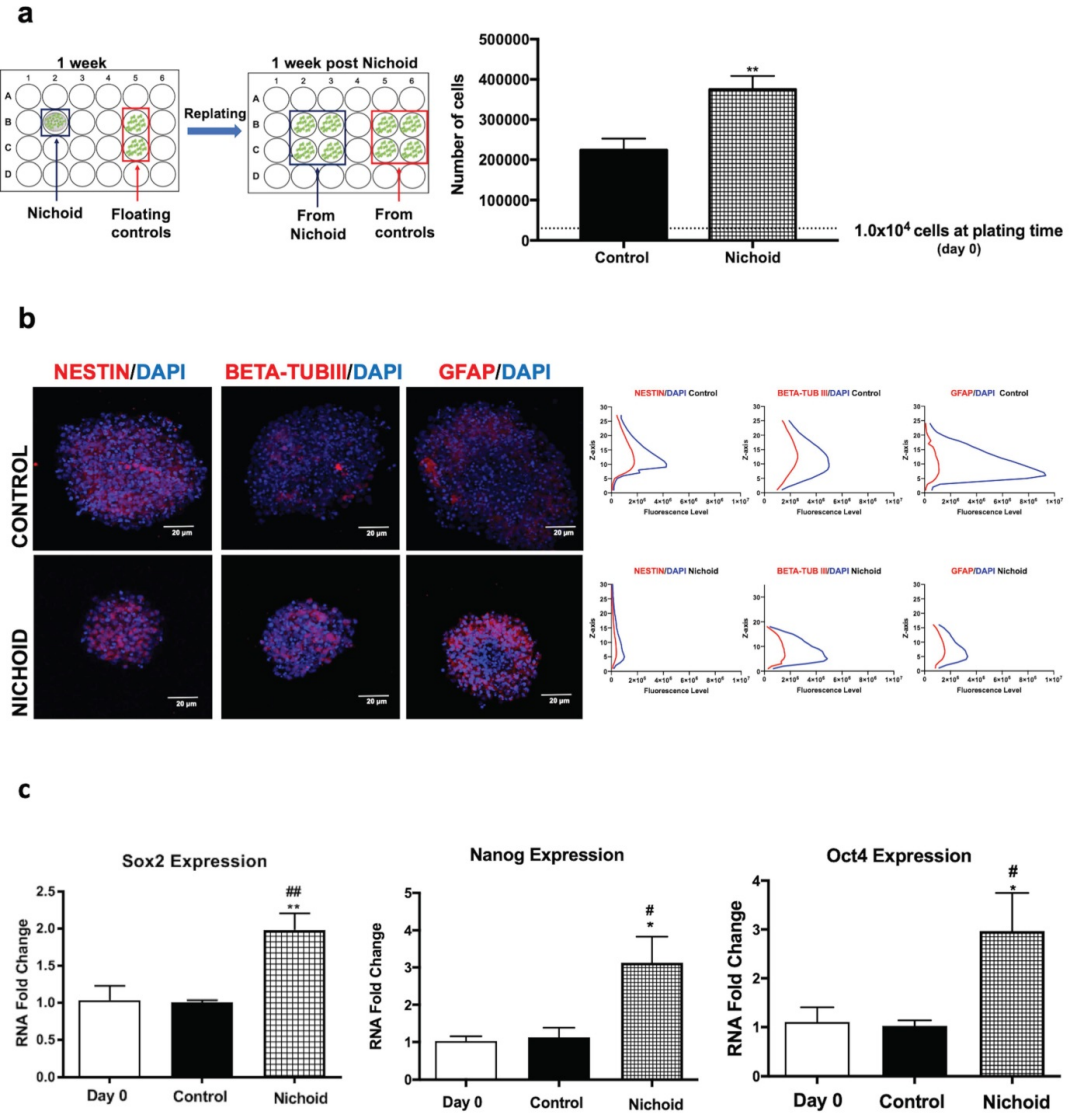

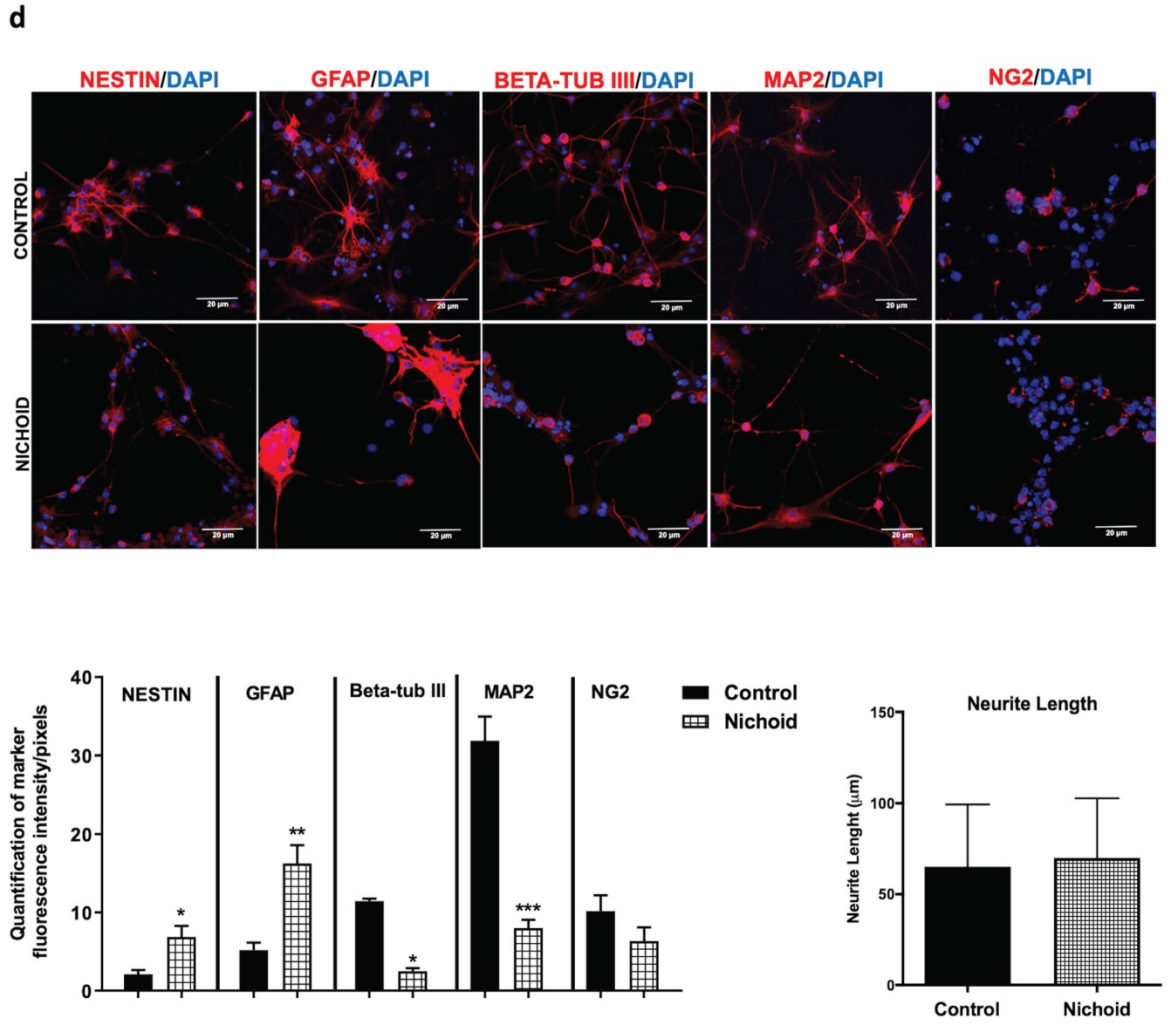
** NPCs detached from the Nichoid retain a memory of pluripotency induction. A.** NPCs were grown in standard floating conditions or inside the 3D scaffold for 7 days, then replated (1x10^4^ cells/well) in standard floating conditions for 7 more days (please see schematic). Cells were counted with trypan blue exclusion method (blind observer). Data are expressed as mean of three independent experiments ± SD (**p <0.01 vs Control). **B.** Representative immunofluorescence images of NESTIN, BETA-TUBIII, GFAP in replated NPCs. Nuclei were stained in blue (DAPI). Scale bar 20 µm. The adjacent panels show the markers' distribution along the Z axis. The quantification of fluorescence intensity was made with ImageJ software. **C.** mRNA expression levels of Sox2, Oct4 and Nanog in replated NPCs. Data are expressed as mean of three independent experiments, each performed in duplicate ± SEM (n=6, *p <0.05, **p <0.01 vs Control; #*p* < 0.05; ##*p* <0.01 vs NPCs at day 0). **D.** Representative immunofluorescence images of NESTIN, GFAP, BETA-TUBIII, MAP2 and NG2 of NPCs grown for 7 days inside the Nichoid and then differentiated for 7 more days. Nuclei are stained in blue (DAPI) and the other markers in red. Scale bar 20 µm. The graphs report the quantification of fluorescence intensity made with ImageJ software. Pictures are representative of three different experiments. Data has been reported as mean ± SD (n=3, * *p* < 0.05, **p <0.01 and ***p <0.001 vs Control). Neurite length made with ImageJ was not significantly different between the two conditions. The data reported in the diagram are referred to mean ± SD.

In order to be able to transplant NPCs *in vivo*, we investigated whether NPCs grown inside the Nichoid maintain the features described above when kept in culture in standard floating conditions after growth in the Nichoid (Figure 1A and Figure 4A). 1×10^4^ cells were plated inside the Nichoid and let grow for 7 days, subsequently they were detached and re-plated in standard floating conditions (Figure 4A and Materials and Methods section). Figure 4A shows that these NPCs almost doubled the number of cells alive with respect to normal conditions, suggesting that they retained a memory of their past growth inside the Nichoid. Neurospheres were counted and divided in different groups according to their dimensions (Figure S5A). The number of spheres with a volume range of 1-5×10^6^ µm^3^ was more abundant with respect to the relative control conditions. On the other hand, the percentage of neurospheres with a volume greater than 1×10^7^ µm^3^ is significantly lower for the cells previously grown inside the Nichoid. This suggests that NPCs after being expanded in the Nichoid present a different three-dimensional organization. On the other hand, the distribution of Nestin, beta-tub III, and GFAP, investigated by immunofluorescence analysis, in neurospheres derived from NPCs expanded inside the Nichoid was found to be comparable to the distribution in controls (Figure 4B). Moreover, beta-tub III and GFAP expression is higher in NPCs previously expanded in the 3D micro-scaffolds (Nichoids) respect to the relative controls. Gene expression of pluripotency markers (Sox2, Nanog and Oct4) in NPCs grown inside the Nichoid and then re-plated in normal floating conditions was investigated by Real Time PCR. The higher expression of these mRNAs is maintained by NPCs after being expanded in the Nichoid compared to those in standard floating conditions (Figure 4C).

The differentiation capabilities of the NPCs after being expanded in the Nichoid were evaluated (Figure S5B). The expression of typical neural markers Nestin, GFAP, beta-tub III, Map2 and NG2 was investigated by immunofluorescence (Figure 4D and Figure S5C). Cells grown inside the Nichoid expressed a higher level of stemness markers Nestin and GFAP with respect to the control (Figure 4D and Figure S4C). On the other hand, the markers of differentiation, beta-tub III, MAP2 and NG2 appeared more expressed in the control with respect to the cells previously expanded inside the Nichoid (Figure 4D and Figure S5C). Moreover, we observed that the neurite's length, after immunofluorescence with MAP2, a cytoskeleton marker, was not significantly different in the two investigated conditions (Figure 4D and Figure S5D).

### The Nichoid enhances the *in vivo* therapeutic efficacy of NPCs

The therapeutic potential and safety of NPCs expanded inside the Nichoid for 7 days was evaluated by their intrastriatal infusion (0.7×10^5^ cells/mouse) in PD affected mice (Figure S6). This was compared to the infusion of NPCs grown in standard floating conditions at the dosage of 2.5×10^5^ cells/mouse, which was the active dosage able to give rise to therapeutic effects in PD animal models 3, 18, 43. The infusion procedure of NPCs was successful, and all transplanted animals remained alive.

**Figure 5:**
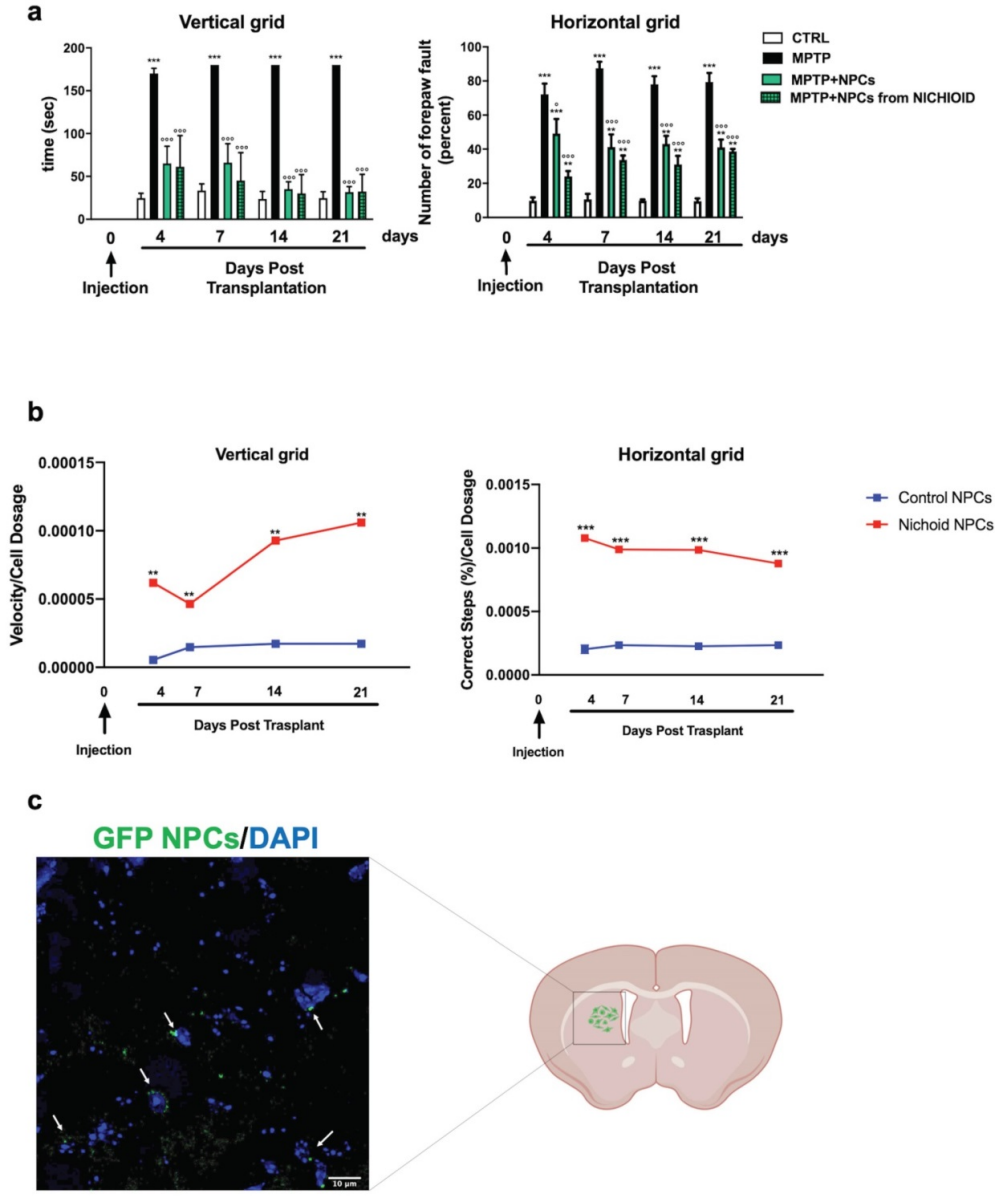
** NPCs expanded inside the Nichoid show increased therapeutic effects promoting recovery of function. A.** Histograms report behavioral analysis (vertical and horizontal grid tests). Please see M&M section for details. Data are expressed as mean ± SD. (n=6; **p <0.01, ***p < 0.001 vs CTRL; °*p* < 0.05, °°°*p* < 0.001 vs MPTP). **B.** NPCs therapeutic effects with respect to cells' dosage. Data are expressed as mean ± SD. (n=6; ***p <0.001 vs Control). **C.** Immunofluorescence image of engrafted Nichoid-grown GFP-NPCs infused in PD animals. Nuclei are stained with DAPI. Scale Bar: 20 µm.

**Figure 6:**
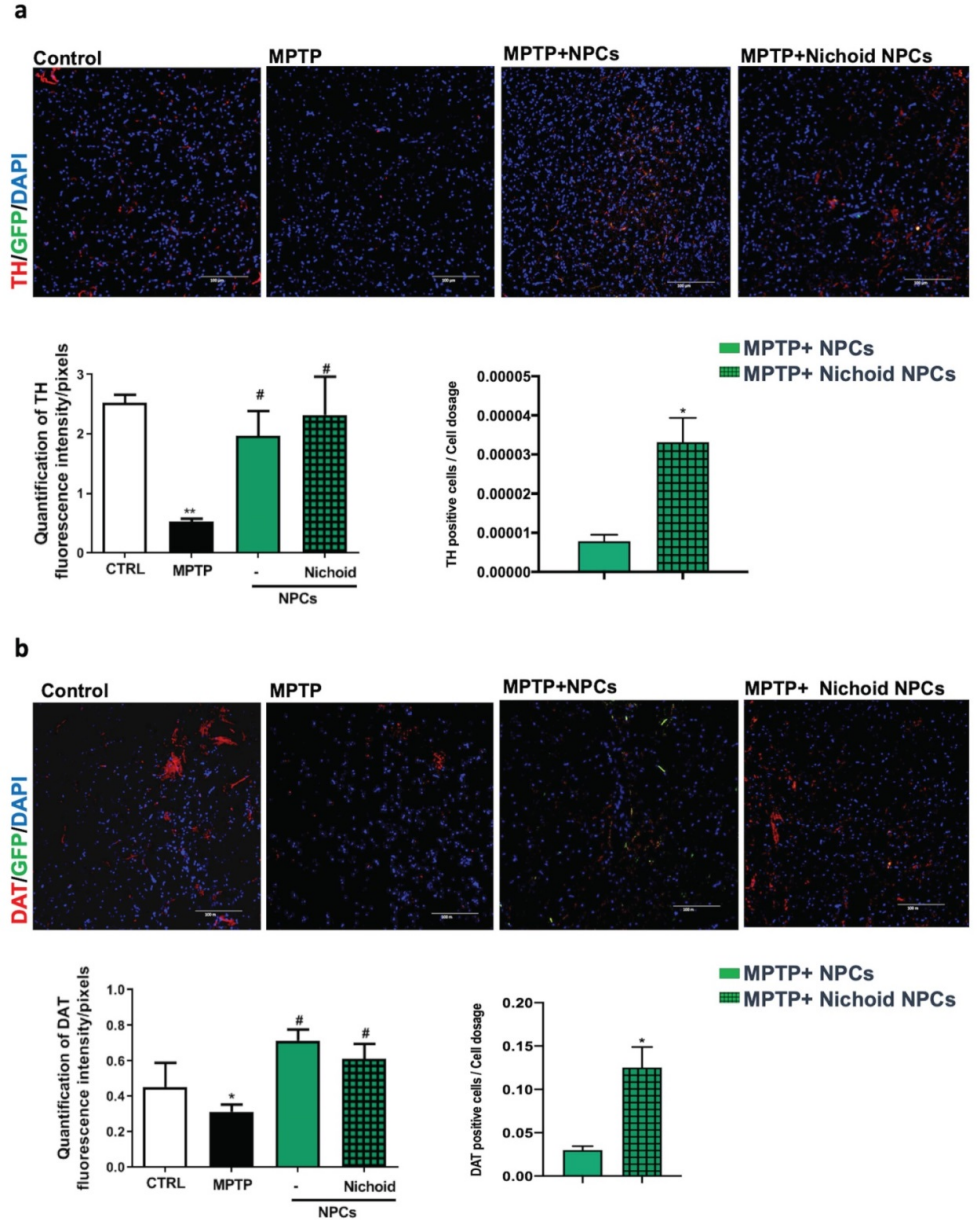

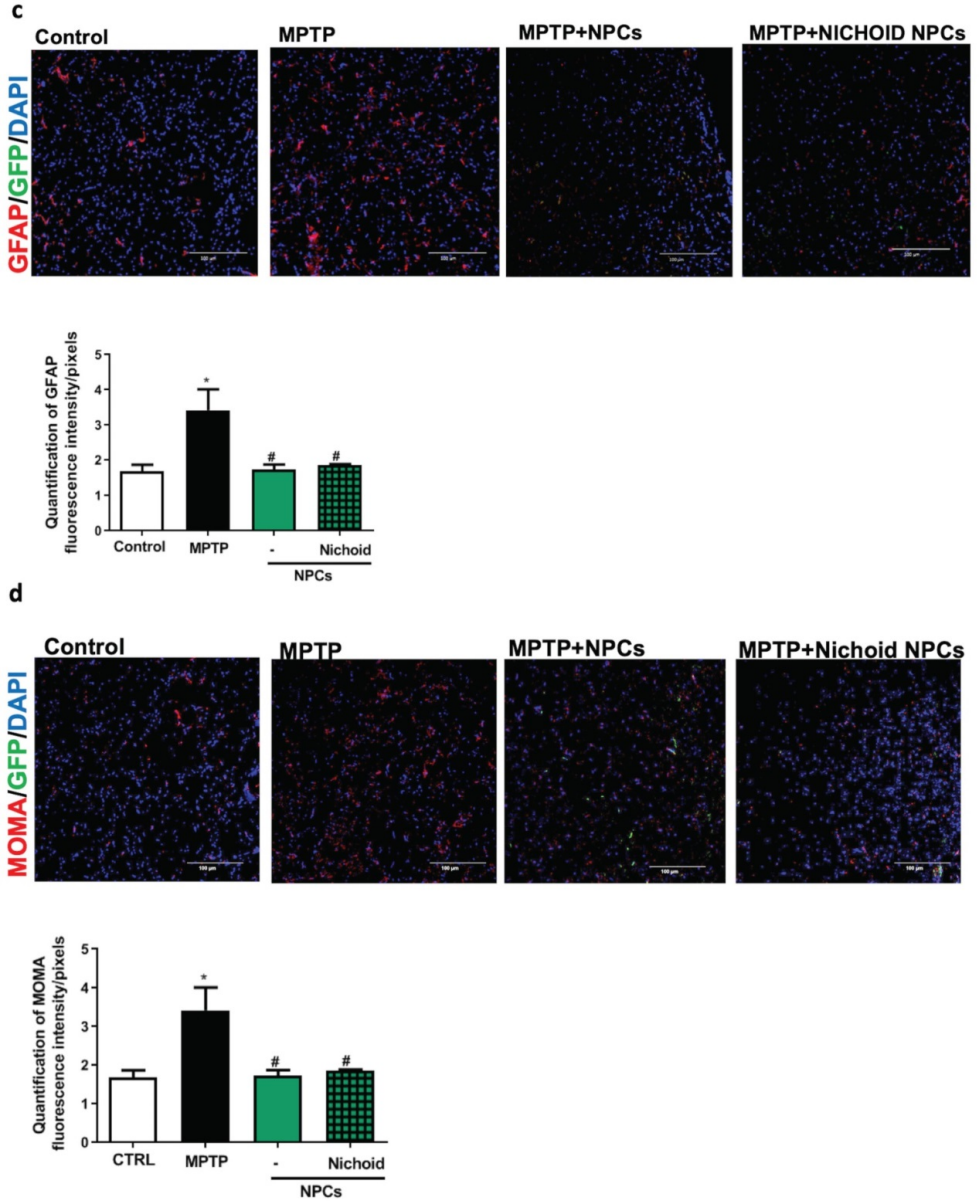
** NPCs expanded inside the Nichoid promote dopaminergic recovery and reduced inflammation. A.** Immunofluorescence images of TH-staining in striatum sections. Nuclei are stained with DAPI. Scale bars=100 µm. The histogram reports TH fluorescence intensity. Data are expressed as mean ± SD (n=3; **p <0.01 vs Control, #*p* <0.05 vs MPTP). The adjacent histogram reports the percentage of positive cells normalized to the dosage of transplanted NPCs. Data are expressed as mean ±SD (n=3; *p <0.05 vs MPTP-NPCs). **B.** Immunofluorescence images of DAT expression in striatum sections. Nuclei are stained with DAPI. Scale bar: 100μm. The histogram reports DAT fluorescence intensity. Data are expressed as mean ± SD (n=3: *p <0.05 vs Control, #*p* <0.05 vs MPTP). **C.** Immunofluorescence images of GFAP expression and morphology in striatum sections. Nuclei are stained with DAPI. The histograms report the quantification of GFAP-fluorescence intensity. Data are expressed as mean ± SD (n=3 *p <0.05 vs Control, #*p* <0.05 vs MPTP). Scale bar: 100 µm. **D.** Immunofluorescence images of MOMA (CD68)-labeled striatum sections. The histogram reports GFAP fluorescence intensity. Data are expressed as mean ± SD (n=3 *p <0.05 vs Control, #*p* <0.05 vs MPTP). Scale bar: 100 µm.

The administration of the neurotoxin MPTP following a specific paradigm causes damages to 70%-80% of the nigrostriatal projections 3, 18, 43. This leads to the increase of the time taken to descend from the grid in the vertical grid test (Figure 5A). Similarly, the forepaw faults of the anterior paw, which can be appreciated in the horizontal grid test, were increased (Figure 5A). The therapeutic effect of NPCs, both grown inside the Nichoids and in standard conditions in suspension, was promising and it favored an enhancement in functional recovery both in the horizontal and in the vertical grid tests (Figure 5A). Moreover, a significant improvement between the conditions is noted when the behavioral performances were normalized to the administrated cell dosages (Figure 5B). The therapeutic effect was already significant 3 days after transplantation and the initial functional recovery observed in animals treated with NPCs grown inside the Nichoid is maintained for the entire observational period (21 days after injection). The attention was then focused on the different dosages of NPCs infused: a better performance was obtained with NPCs grown inside the Nichoid (Figure 5B). Three weeks post transplantation mice were sacrificed, and their brains sectioned 3, 18, 43 observing that transplanted NPCs efficiently engrafted in the striatum (Figure 5C). In brains of transplanted animals' tumorigenic cells were not found and engrafted NPCs were not proliferating as indicated by the ki67 staining, a marker of active proliferating cells 63, 64 (Figure S7). MPTP causes a massive loss of Tyrosine Hydroxylase (TH)-positive cell bodies and axons that was quantified to be over 70% 3, 18, 43. The protective action of infused NPCs was assessed investigating this dopaminergic marker in the engrafted striatum. By performing quantitative confocal laser microscopy on striatal sections, it was found that NPCs significantly counteracted the MPTP-induced loss of TH-positive innervations (Figure 6A). The quantification of striatal TH expression shows that the recovery of this dopaminergic marker was higher in mice treated with NPCs expanded inside the Nichoid with respect to the infusion of NPCs grown in standard floating conditions (Figure 6A). In line with this evidence, the striatal expression of the dopamine transporter (DAT or SLC6A3) (Figure 6B), was found to be restored in the striatum of mice treated with NPCs grown in the Nichoid and control NPCs versus MPTP parkinsonian animals (Figure 6B). Inflammation is part of the physiological response to neurodegeneration as indicated by astroglia activation 3, 18. Figure 6C and 6D show respectively that the density of reactive GFAP-positive cells and CD68 (MOMA)-positive cells was markedly reduced in the striatum of mice treated with Nichoid-cultured NPCs with respect to the MPTP mice (Figure 6C and 6D).

## Discussion and Conclusion

Here we demonstrate that the expansion of NPCs in the Nichoid miniaturized scaffold, mimicking the 3D structural properties of the stem cell niche, promotes pluripotency and potentiates the efficacy of these cells in an experimental animal model of PD. By employing a large culture surface and Nichoid coverage (we used 170 samples of 0.5 cm^2^/each, covered by Nichoids by 90%), we were able to culture almost all the cells inside the 3D Nichoid and to harvest millions of cells for the study. In this regard, the use of a multi-foci approach allowed us to reduce the Nichoid fabrication time down to 2.5 h/sample 29. As a result of a ten years long technological effort in the up-scaling of the laser micro-fabrication technique, we are now able to obtain enough expanded cells from the Nichoid substrate to further increase the robustness and the quality of our observations. This achievement may open an avenue towards the production of clinically significant cell doses, in view of the translation of SC-based therapies.

We observed that adult stem cells grown inside the Nichoid not only remain more viable than the controls but that the Nichoid is capable of inducing pluripotency. Our micro-scaffold interacts with cells at a microscopic level, conditioning the spatial adhesion of cells while significantly reprogramming specific signaling pathways which regulate pluripotency, in the absence of exogenous chemical inductive factors. This allows us to obtain a pluripotency enhancement induced exclusively by the structural properties of the scaffold, i.e. the 3D geometry and stiffness, without operating any chemical and/or genetic induction on the cells. We also showed here that, thanks to the peculiar structural characteristics of the Nichoid, the cells that proliferate inside it undergo conditioning by the physical constraints provided by the scaffold lattice and are induced to organize themselves into aggregates with an internal core surrounded by an external wall, similarly to what is observed in neurospheres. Moreover, from an analysis on the distribution of markers in the different levels of the scaffold, the peak of expression for SOX2, OCT4 and NANOG was around 10-15 µm, suggesting a possible co-localization with NESTIN marker, differently from beta-tub III distribution.

The substantial difference is to be found in the fact that the geometry of the Nichoid prevents the aggregates that are created within its lattice from increasing in diameter beyond a certain limit, precisely imposed by the Nichoid's architecture. Thanks to this growth threshold, the cellular core enhances its proliferative capacity, instead of reducing it due to diffusive barriers as in neurospheres culture. Most importantly, said induction is maintained by the cells even after they are detached from the Nichoid and put back in culture under standard conditions, i.e. in neurospheres, suggesting a stable modification of the pathways involved. In essence, we obtained the reprogramming of pluripotency by purely controlling the structural interactions of the cell with their adhesion microenvironment, coherently with recent demonstration that stiffness is increased in the neurogenic niches of the brain 65. This is also relevant and in agreement with recent findings highlighting the importance of xeno-free biomaterials composition in SCs culture *in vitro* 66, 67.

Few studies investigated the potentiality of scaffolds and SCs in the treatment of Parkinson's Disease (PD) 7, 68, 69. Carlson and colleagues developed three-dimensional microtopographic scaffolds using tunable electrospun polymeric substrates with pluripotent stem cell-derived neurons 69, 70. In their work these authors showed that the transplantation of scaffold-supported neuronal networks into the striatum of mouse brain improved the cells survival 69, 70. Moreover, biomaterials are more implicated in axonal guidance or neuronal migration in order to reconstruct degenerated neural circuits 71-74. Alternatively, the delivery of pharmacological agents or cells can be potentiated with hydrogels 75. To our knowledge, our work is the first that combine the 3D micro-niche Nichoid and SCs for a therapeutic efficacy in PD preclinical model as the cells therapeutic effect has never been tested. Here, in an experimental animal model of PD, we demonstrate that Nichoid-expanded NPCs have an improved efficacy compared to NPCs cultured in conventional spheroids. Our results demonstrate that not only the transplanted cells are safe, but their therapeutic dose can be reduced by two thirds with the same efficacy, compared to spheroid-cultured cells. The main obstacle to SCs translation into clinical practice is the lack of specific *in vitro* culture methods able to guarantee large-scale expansion while maintaining safety, potency and function 36. Our findings may have a tremendous impact in the clinics, opening a new perspective of production of large amounts of safe and functional NPCs for use in regenerative therapies of the nervous system. If confirmed for other SCs types, these results could highly impact the broader field of regenerative medicine.

## Supplementary Material

Supplementary figures and table 1.

Supplementary table 2.

## Abbreviations

Embryonic stem cells: ESCs
environmental scanning electron microscopy: ESEM
green fluorescent protein: GFP
leukemia inhibitor factor: LIF
Kyoto Encyclopedia of Genes and Genomes: KEGG
mesenchymal stem cells: MSCs
neural progenitor cells: NPCs
Parkinson's disease: PD
stem cells: SCs
three dimensional: 3D
two-photon laser polymerization: 2PP
1-methyl-4-phenyl-1,2,3,6-tetrahydropyridine: MPTP
