# Supplementary material for: Neural precursors cells expanded in a 3D micro-engineered niche present enhanced therapeutic efficacy in vivo
Source: Nanotheranostics. 2021 Jan 1;5(1):8–26. doi: 10.7150/ntno.50633 (PMC7738947; doi:10.7150/ntno.50633)
Supplement: Supplementary file 1 — Supplementary figures and table 1. [file ntnov05p0008s1.pdf]

SUPPLEMENTARY FIGURES

<u>SUPPLEMENTARY FIGURE 1</u>

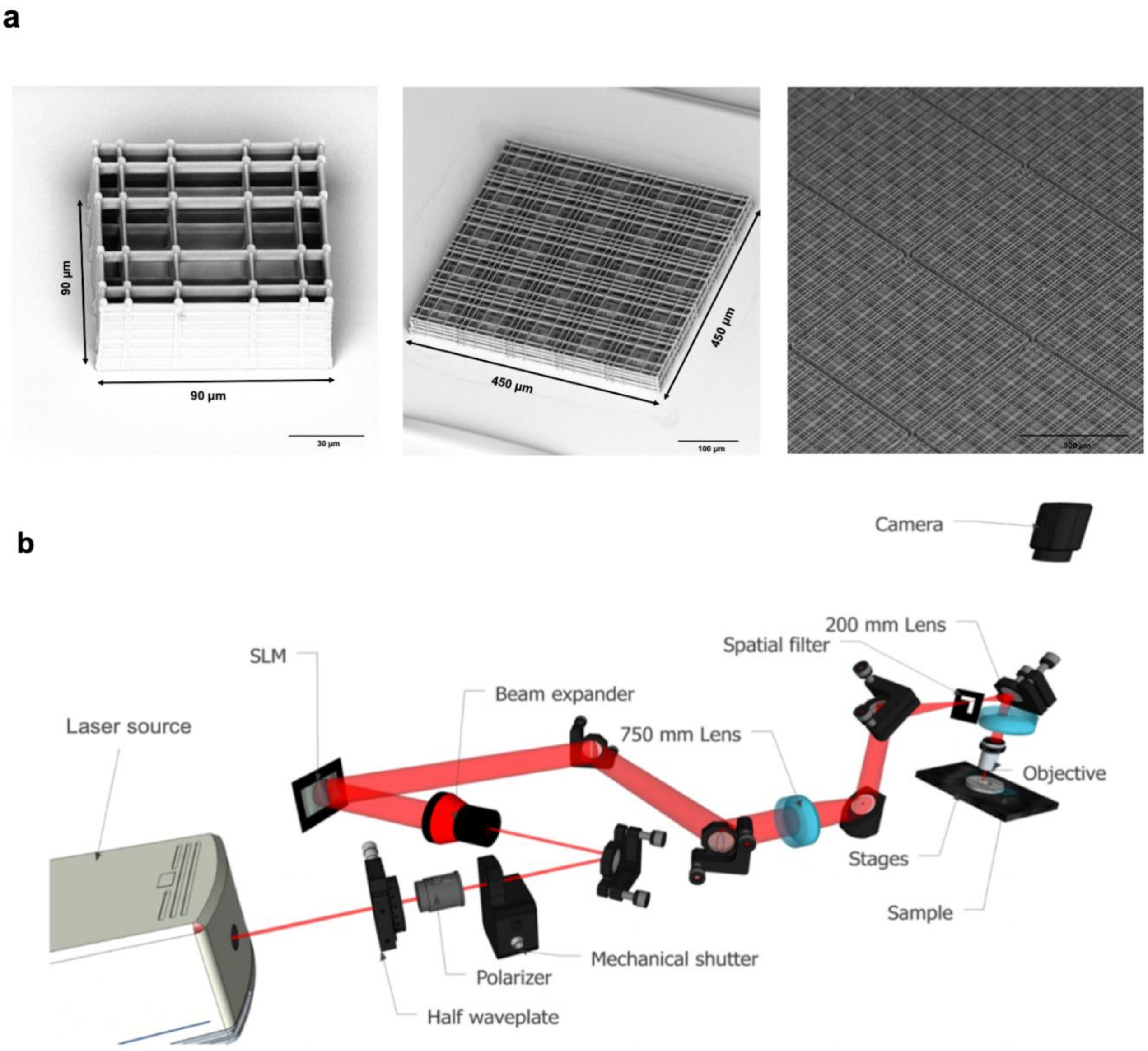

<u>SUPPLEMENTARY FIGURE 1. Nichoid scaffold fabrication</u>

A) Nichoids' quality control images obtained by Scanning Electron Microscopy (SEM) at 5kV. SEM images of: a single niche (Scale bar: 30µm), a block of 5x5 of single niches (Scale bar: 100µm), and a supermatrix of niches (Scale bar: 300µm). The images show a representative example of a good manufacture result used for biological experimental validation.

B) Schematic representation of the new optimized set-up for the multi foci laser polymerization composed by femtosecond laser source, a beam expander, a 4f telescope and a Spatial Light Modulator (SLM).

SUPPLEMENTARY FIGURE 2

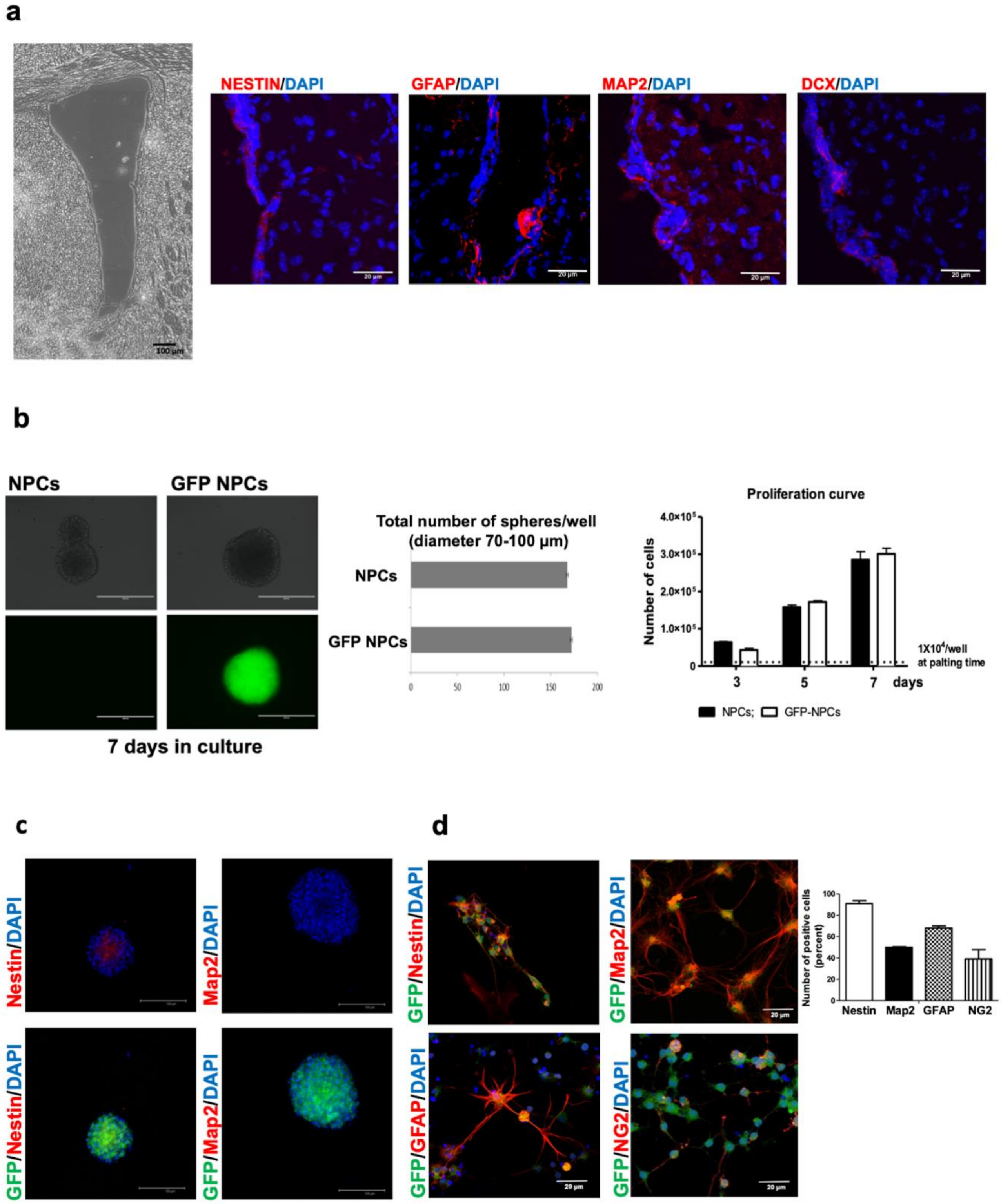

SUPPLEMENTARY FIGURE 2. Characterization of NPCs obtained from the SVZ

A) Representative direct live image of the Sub Ventricular Zone (SVZ) from where NPCs were isolated. Scale bar 100 µm. The adjacent panels show representative SVZ-immunofluorescence characterization evaluating the expression of NESTIN (red), MAP2 (red), GFAP (red), DCX (red) and DAPI for nuclei (blue). Scale bar 20 µm.

B) Representative direct live images of NPCs and GFP NPCs after 7 days of culture in floating conditions. Scale bar 200 µm. The histograms report the evaluation of the number of spheres with a dimension comprised between 70-100 µm diameter and the proliferation curve respectively of NPCs and GFP NPCs seeded at a starting density of $1x10^4$ cells. Cells were counted in triplicate with trypan blue exclusion method after 3, 5, and 7 days. Data are reported as mean ± SD.

C) Representative immunofluorescence images of neurospheres [NPCs (top panels) and GFP NPCs (lower panels)] expressing NESTIN (red) and MAP2 (red). The lower panels also show co-staining of GFP positive cells (green). DAPI (blue) was used for nuclei staining. Scale bar 100µm.

D) Representative immunofluorescence images of GFP NPCs differentiated for 7 days (See Materials and Methods section for further details). Cells were characterized by evaluating the expression of NESTIN (red), MAP2 (red), GFAP (red) and NG2 (red). Nuclei were labeled in blue (DAPI). The histogram reports the percentage of GFP NPCs positive to the respective markers. Data are expressed as mean of the quantification performed in three different fields for each condition ± SD (n=3). Scale bar 20 µm.

SUPPLEMENTARY FIGURE 3

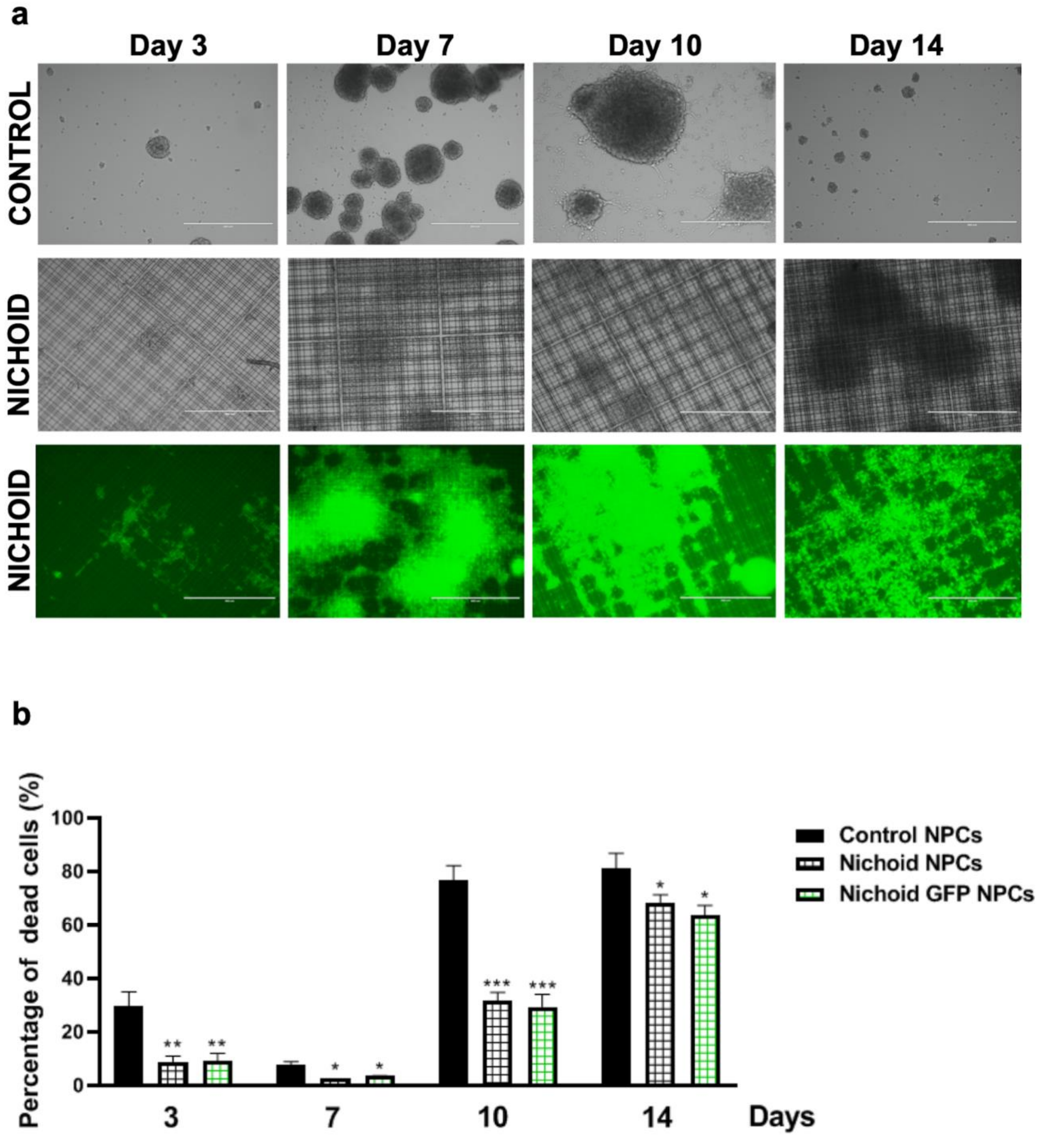

SUPPLEMENTARY FIGURE 3. Proliferation of NPCs expanded inside the Nichoid

A) In vivo direct light images (EVOS FL microscope, Euroclone) of NPCs neurospheres maintained in stem cells medium in standard floating conditions (CONTROL) or grown inside the Nichoid (NICHOID) at day 3, 7, 10 and 14. The images refers to NPCs and GFP NPCs.

Scale bar 400 μm. Images are representative of what was observed in at least three different independent experiments.

B) Percentage of dead cells at different time points of NPCs and GFP NPCs grown inside the Nichoid and in standard floating condition. Cells were plated in NSC medium at the density of $1x10^4$ cells at plating time. Cells were counted with trypan blue exclusion method after 3, 7, 10, 14 days. Data are expressed as mean of at least three independent experiments ± SD (n=3). Statistical analysis was performed with Student's t- test followed by Bonferroni post-test (***$p<0.001$ vs Control NPCs).

SUPPLEMENTARY FIGURE 4

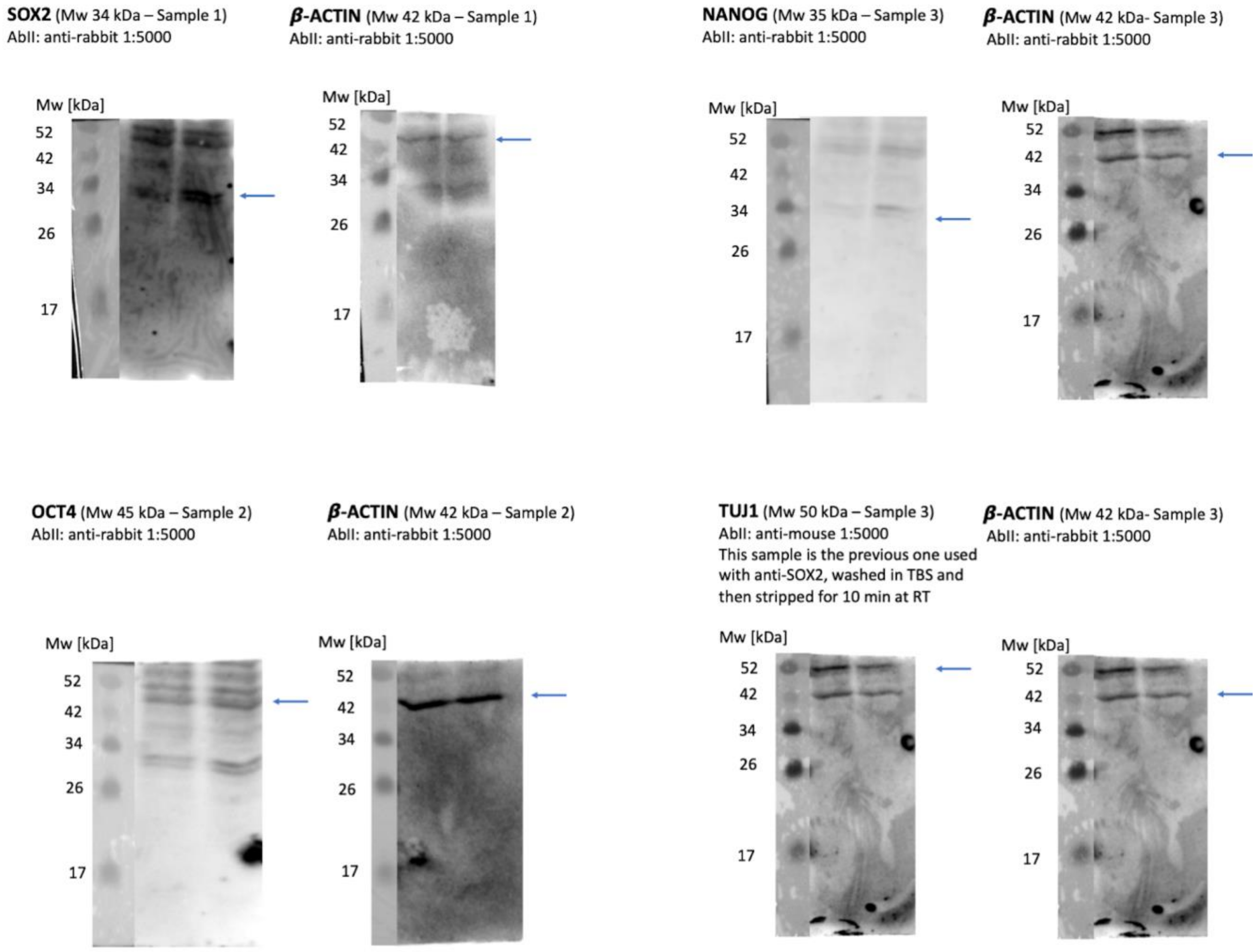

SUPPLEMENTARY FIGURE 4. Full blots of Western Blot analysis.

SUPPLEMENTARY FIGURE 5

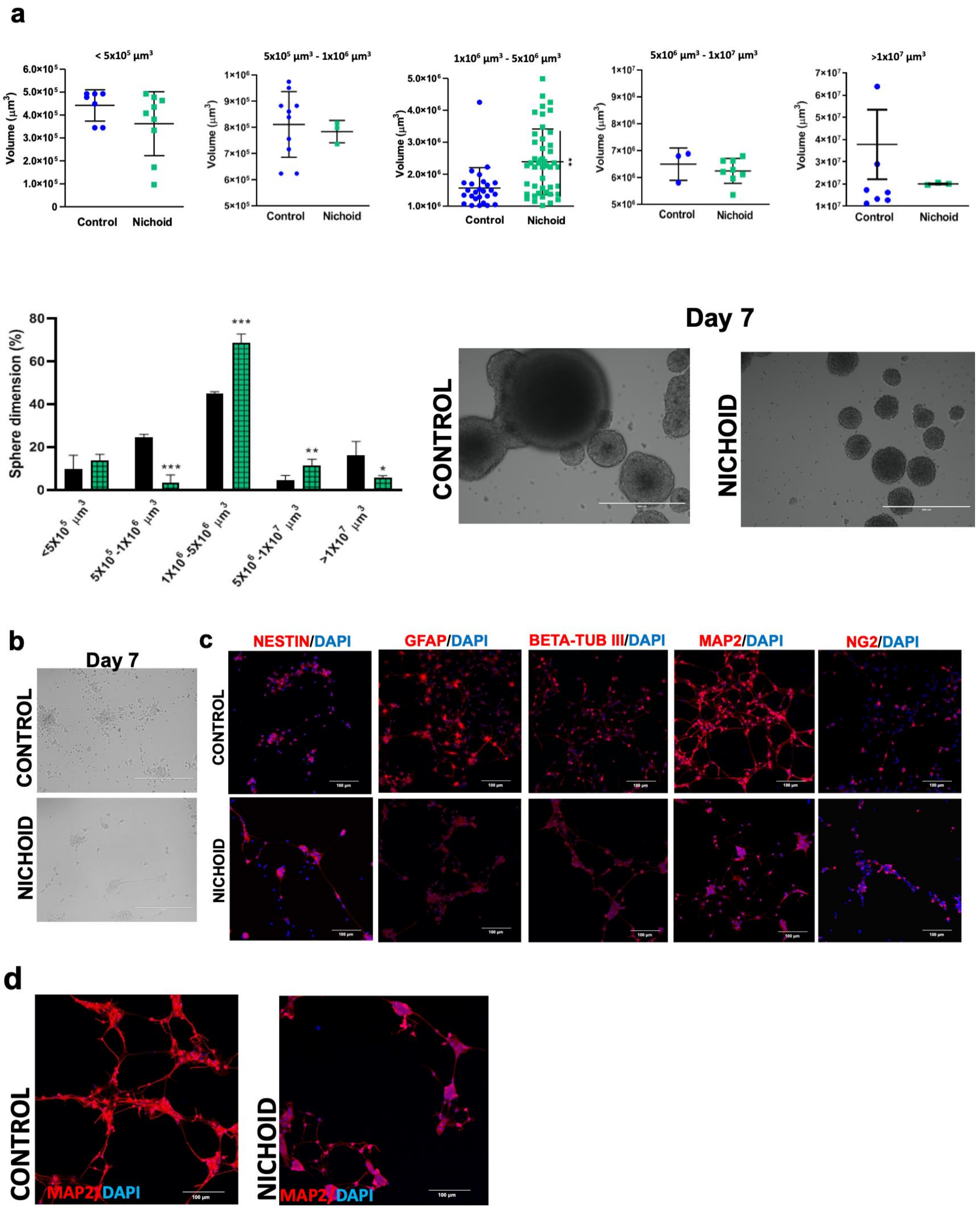

SUPPLEMENTARY FIGURE 5. Structural organization and differentiation capabilities of NPCs replated after Nichoid-growth

A) The box plots report the different dimension of the neurospheres grown inside the Nichoid and in control floating conditions. The histogram reports the percentage of spheres dimension distribution (*$p < 0.05$ vs Control, **< 0.001 and ***$p<0.001$ vs Control). Representative direct light images of neurospheres obtained from NPCs grown inside the Nichoid and then replated in standard floating condition ($1x10^4$ cells at plating time). Scale bar: 400 µm.

B) Representative direct light images of NPCs expanded inside the Nichoid for 7 days and then differentiated in standard conditions for 7 more days. Cells were plated at the density of $1x10^4$/well. Scale bar: 200 µm.

C) Representative immunofluorescence images of NESTIN, GFAP, BETA-TUBIII, MAP2, and NG2 expression in NPCs grown for 7 days inside the Nichoid and then differentiated for 7 more days. Nuclei are stained in blue (DAPI) and the other markers are in red.

D) Representative immunofluorescence images of MAP2 expression in NPCs grown for 7 days inside the Nichoid and then differentiated for 7 more days in standard conditions. Nuclei are in blue (DAPI) and MAP2 is shown in red. Scale bar 100 µm.

SUPPLEMENTARY FIGURE 6

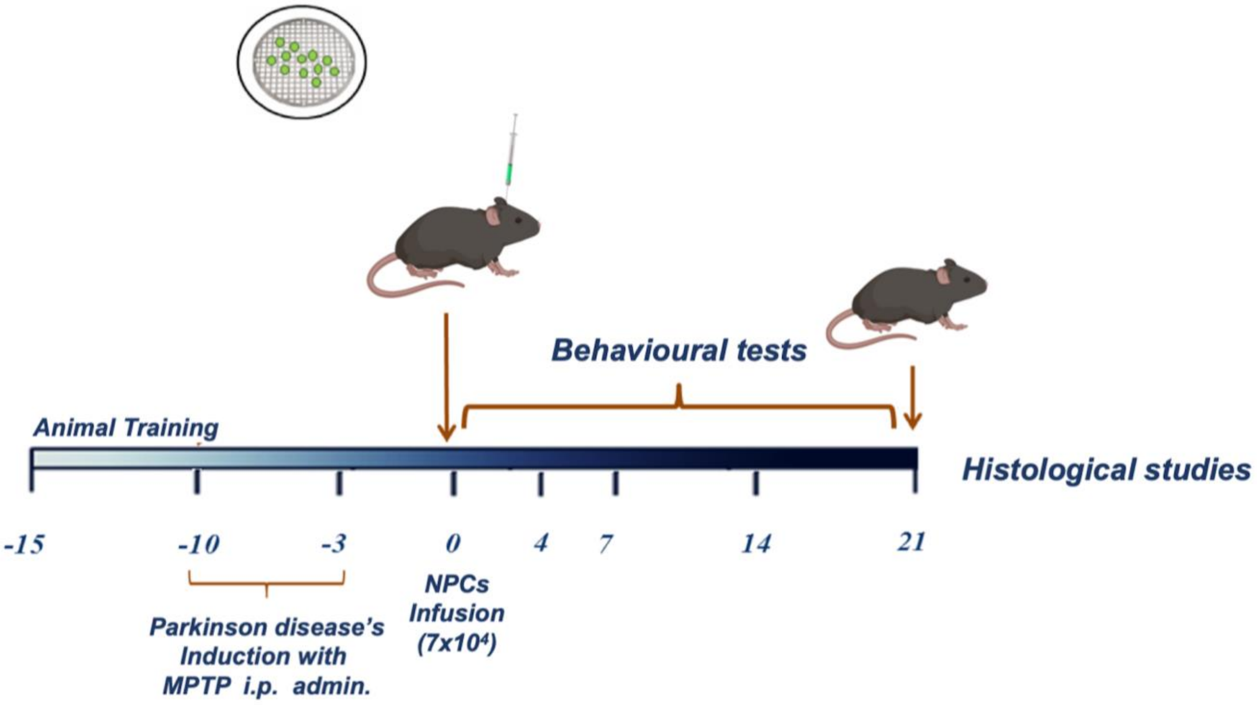

SUPPLEMENTARY FIGURE 6. Experimental set up of in vivo experiments. Experimental set up to investigate in vivo the therapeutic effects of NPCs grown inside the Nichoid.

SUPPLEMENTARY FIGURE 7

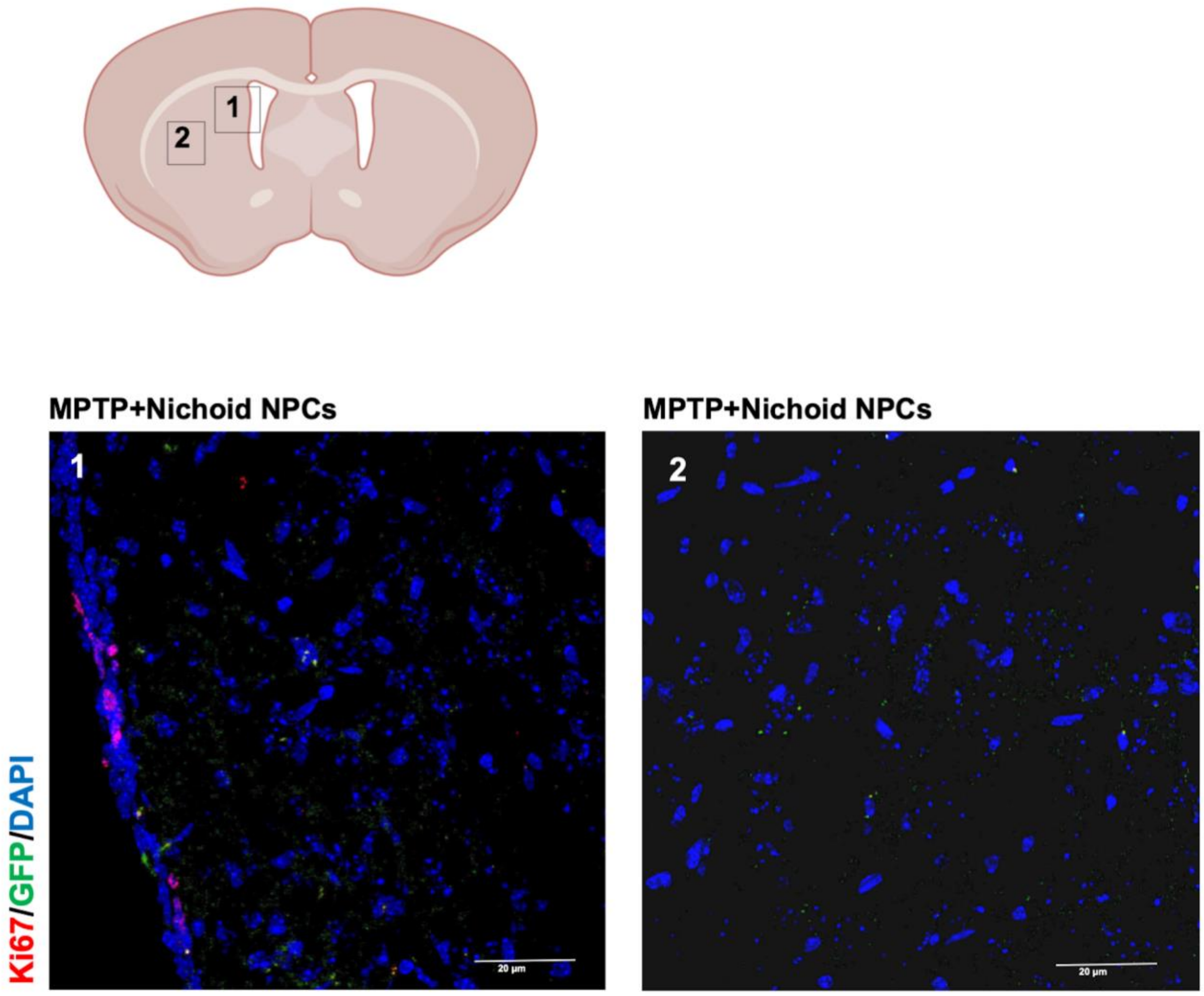

SUPPLEMENTARY FIGURE 7. Transplanted NPCs do not show tumorigenic potential

Representative immunofluorescence images of ki67 expression in striatal coronal sections of MPTP injected mice treated with Nichoid expanded NPCs. Ki67 is shown in red, Nuclei are in blue (DAPI), green refers to engrafted GFP NPCs Scale bar: 20 µm.

SUPPLEMENTARY TABLE 1. List of Primers used for Real Time PCR

Sox2 FW	CATGTATAACATGATGGAGACGGAGCTGAA
Sox2 REV	TTACGCCGCTGCCCCCGGGACCATACCATG
Oct4 FW	AAGGGCCTCCAGGTGGGCCTGGAATCGGAC
Oct4 REV	CTCGTGCTCCTGCCTGGCCCTCAGGCTGCA
Nanog FW	GACAAGGGCCCTGAGGAGGAGGAGAACAAG
Nanog REV	TTATAGCTCAGGTTCAGAATGGAGGAGAGT
Nestin FW	GGAGGCTGAGAACTCTCGCTTGCAGACACC
Nestin REV	TATTAGGCAAGGGGGAAGAGAAGGATGTTG
Beta-tub III FW	TTCACCACCAGCGCCAGCACCCCTGTGACC
Beta-tub III REV	AATGTTCACGGAAGTGGCCGTGCTTGGGAG
c-Myc FW	CAGCGACTCTGAAGAAGAGCA
c-Myc REV	TTGTGCTGGTGAGTGGAGAC
Fgf2 FW	AGTGCTCTGTGCAGAAGTGT
Fgf2 REV	TCTGTCCAGGTCCCGTTTTG
Smad3 FW	AAGAAGCTCAAGAAGACGGGG
Smad3 REV	CAGTGACCTGGGGATGGTAAT
Gapdh FW	CCAGGGCTGCCATTTGCAGTGGCAAAGTGG
Gapdh REV	CCTGGAAGATGGTGATGGGCTTCCCGTTGA.

SUPPLEMENTARY TABLE 2. Deregulated genes involved in pluripotency

Genes were ranked according to their fold change expression in Nichoid versus Control conditions.
